# High-Performance Twisted Nylon Actuators for Soft Robots

**DOI:** 10.34133/research.0642

**Published:** 2023-03-17

**Authors:** Jin Sun, Shijing Zhang, Jie Deng, Jing Li, Dong Zhou, Dehong Wang, Junkao Liu, Weishan Chen, Yingxiang Liu

**Affiliations:** State Key Laboratory of Robotics and Systems, Harbin Institute of Technology, Harbin 150001, China.

## Abstract

Twisted nylon actuators (TNAs) are widely recognized in soft robotics for their excellent load-to-weight ratio and cost-effectiveness. However, their limitations in deformation and output force restrict their ability to support more advanced applications. Here, we report 3 performance-enhancing strategies inspired by the construction process of chromosome, which are validated through 3 novel types of TNAs. First, we design a dual-level helical structure, demonstrating remarkable improvements in the deformation (60.2% vertically and approximately 100% horizontally) and energy storage capability (launching a miniature basketball to 131 cm in height). Second, we present a parallel-twisted method, where the output force of TNAs reaches 11.0 N, achieving 12.1% contraction under a load of 15 N (10,000 times its weight). Additionally, we construct the dual-level helical structure based on parallel-twisted TNAs, resulting in a 439.7% improvement in load capability. We have adopted TNAs for several applications: (a) two bionic elbows capable of rotating and shooting a miniature basketball over 130 cm; (b) a robot that can rapidly jump over 30 cm; and (c) a soft finger that achieves contracting (15.3% contraction under 2 kg load), precise bending (tracking errors less than 2.0%), and twisting motions. This work presents approaches for fabricating high-performance soft actuators and explores the potential applications of these actuators for driving soft robots with multifunctional capabilities.

## Introduction

Soft actuators serve crucial roles in bionic robots due to their compact structure, higher energy density, and more considerable deformation, comparable to rigid actuators. They exhibit diverse structural forms, allowing them to produce longitudinal contraction–extension, bending, and torsional deformations [1–4]. They have been extensively used in driving soft manipulators [5–9], biomimetic robotic hands [10–12], sensors [13–16], and small mobile robots [17–20]. The widespread application of soft actuators aims to enhance the integration of robotic systems and improve safety in human–machine interactions, thereby putting forward high demands on the performance and functionality of these soft actuators.

Currently, the most commonly used soft actuators mainly include pneumatic actuators (PAs) [21,22], dielectric elastomer actuators (DEAs) [17,23,24], and hydraulically amplified self-healing electrostatics (HASELs) [25–27]. PAs are widely used, with many studies focusing on performance improvements through structural optimization. However, the requirement for an additional external air source adds complexity and weight to the system. DEAs are driven by an electric field and require high voltages with high operating frequencies, making them more suitable for mobile robots rather than manipulation tasks. HASELs also require high voltages and necessitate expensive power supply equipment. Additionally, some electrothermally driven soft actuators, such as twisted actuators (TAs) [28–36], have been developed for driving bionic robots, exhibiting excellent output linearity and requiring only a few volts for actuation. Among them, TAs made from nylon fibers (TNAs) achieve a direct rise in temperature through the tightly wrapped electric heating wires and can produce linear contraction. The spring-shaped structure of TNAs originates from the carbon nanotube artificial muscles reported by Lima et al. [3], which achieve a 3% contraction at 1,200 cycles/min but are costly. To address the cost issue, Haines et al. [1] utilized nylon monofilament fibers to fabricate a cost-effective TA, achieving 12% deformation under a load of 0.5 kg. To improve the deformation, Mu et al. [33] proposed a TNA with a large mean diameter and spring index, but the load force decreases to 1 mN, limiting its capability to actuate soft robots. To enhance the output force, Simeonov et al. [37] reported a strategy of weaving multiple TNAs together. However, thermal concentration occurs where multiple TNAs come into contact, making precise control challenging.

**Fig. 1.**
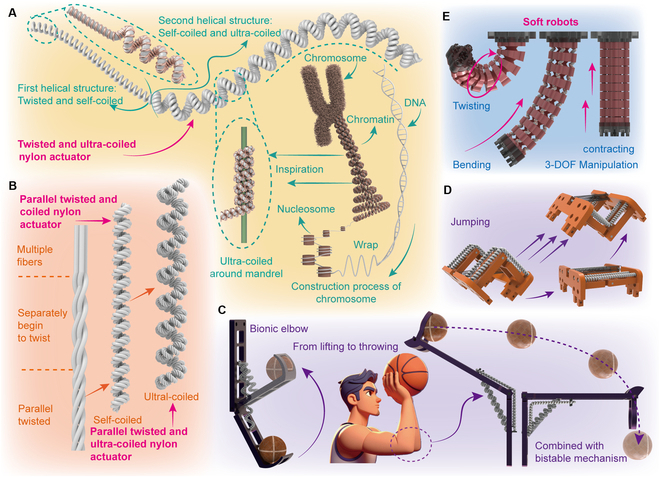
The conception of the high-performance twisted nylon actuators and their applications for actuating soft robots. (A) The process of constructing twisted, self-coiled, and ultra-coiled structures inspired by the chromosomes. (B) The process of constructing a parallel twisted and ultra-coiled structure. (C) Schematic of the bionic elbow from lifting to throwing. (D) Schematic of a small jumping robot that can jump over 15 times the body height. (E) Schematic of a 3-DOF soft finger achieving contracting, bending, and twisting motions.

In summary, the main challenge in simultaneously enhancing the deformation and output force of TNAs arises from their spring-like structure. The deformation of TNAs is determined by their structural stiffness: increasing the diameter enhances deformation but also reduces stiffness, thereby decreasing the output force. Achieving both large deformation and high output force with a single strategy remains challenging. This work presents strategies to separately improve the deformation and output force of TNAs, and then combines them to realize both larger deformation and higher output force simultaneously. Additionally, we further explore their applications in soft robots. The contributions of this work can be summarized as follows: (a) We design a dual-level helical structure for TNAs through the ultra-coiled fabrication process; the deformation reaches 60.2% vertical and approximately 100% in horizontal direction, while providing external energy storage capability. This design is inspired by chromosomal organization, where DNA is coiled multiple times and compacted into a nucleus (Fig. 1A). (b) We propose a parallel-twisted method based on pre-twisted nylon fibers to fabricate a stronger fiber to improve the output force of TNAs to 11.0 N (Fig. 1B). (c) We construct a dual-level helical structure combined with the parallel-twisted method, further improving the output force of TNAs with a dual-level helical structure (increased by 439.7%). (d) The 3 novel TNAs validate these performance-enhancing strategies, covering a broad range of large deformations and high output forces. They are utilized to drive some small bionic soft robots (Fig. 1C and D), enabling substantial lifting of objects (rotation of 102.0°), rapid shooting (over 130 cm), and jumping (15 times body height), which are challenging for thermally driven soft actuators. They are also used in a soft finger (Fig. 1E) that enables contracting (15.6% under 2 kg), precise bending (tracking errors less than 2.0%), and twisting motions. Our strategies are first proposed for TAs made of nylon monofilament fiber; this work substantially enhances the performance of TNAs and explores the multifunctional potential applications in robotics.

## Results

### Deformation improvement mechanism based on the dual-level helical structure

**Fig. 2.**
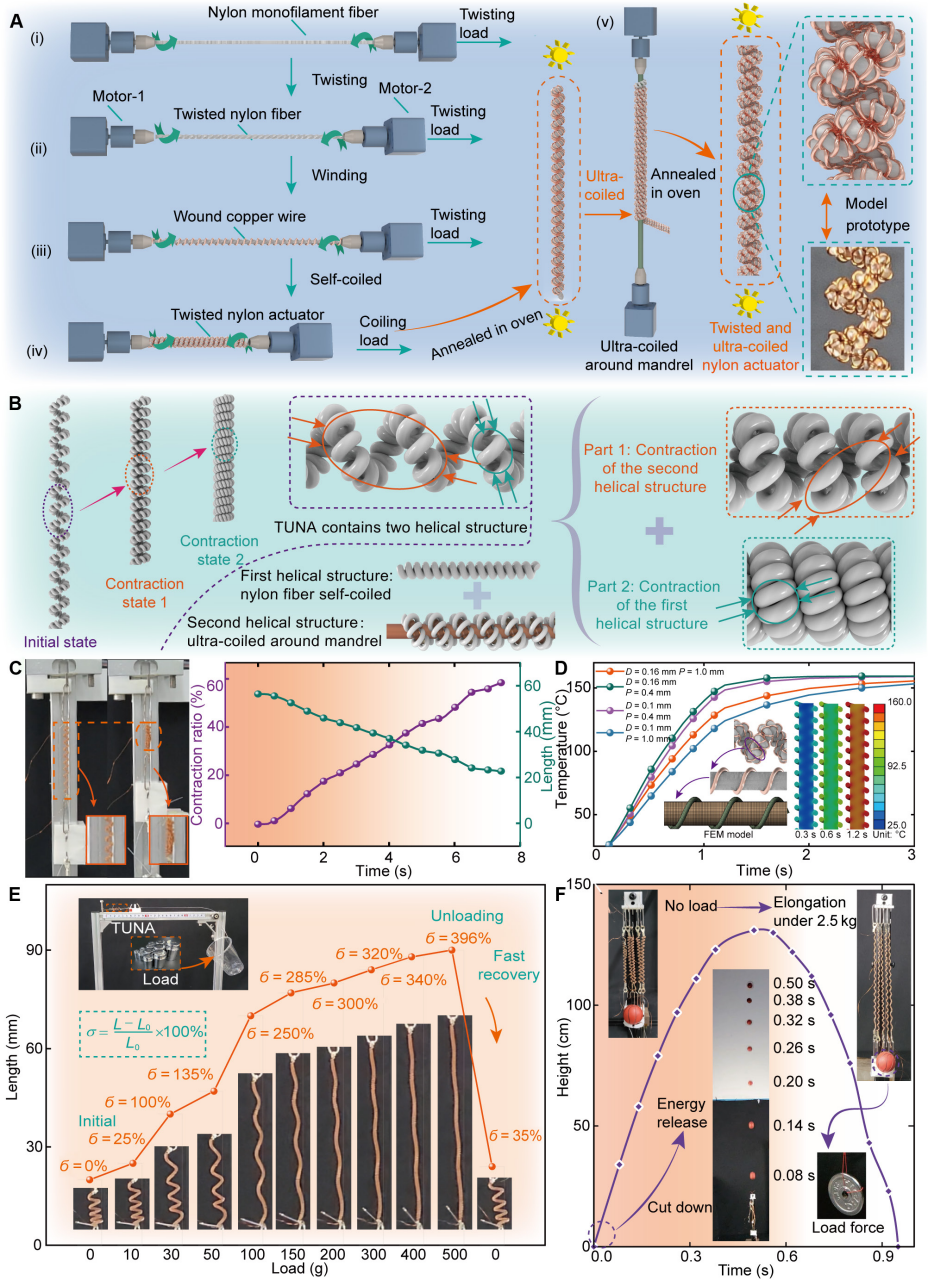
Design and experimental results of the TUNA. (A) The fabrication process of TUNA. (B) Deformation improvement principle. (C) Deformation process of the TUNA. (D) Finite element simulation results of the heat transfer process with different copper wire diameters and winding pitches. (E) Stiffness characteristic of the TUNA when a load of 500 g is gradually added. (F) Energy storage capability. A load of 2.5 kg is applied at the slider, causing the TUNAs to elongate. Then, the load is released suddenly, and the TUNAs contract rapidly, converting the elastic potential energy into kinetic energy.

We initially demonstrated the fabrication process of the twisted and ultra-coiled nylon actuator (TUNA), which has a dual-level helical structure and consists of low-cost nylon fibers and copper wires (see Fig. 2A and Movie S1). The first step involves twisting the nylon fiber in a counterclockwise direction. After that, the copper wires are uniformly wound around the twisted fiber, with the twisting continuing until the fiber self-coiled to form the first-level helical structure. We then coil it around a mandrel to construct the ultra-coiled structure; this approach is necessary because applying additional torsion after the self-coiled process leads to the breakage of nylon fibers. The ultra-coiled structure is formed by coiling in the counterclockwise direction, consistent with the twisting direction of the nylon fiber. Finally, we place the TUNA into a high-temperature drying oven for annealing to eliminate internal stress and lock the helical structure. In the annealing process, the temperature is set to 210 °C for 30 min. The heating rate of the drying oven is approximately 36 °C/min and then cooled to room temperature under natural convection. These steps are finished by self-customized fabrication equipment (see Fig. S1 and Note S1). In contrast to the single-helical structure of TNA, TUNA exhibits a dual-helical configuration. The inner helical structure is formed by self-coiled nylon fibers, and the external helical structure results from the further ultra-coiling around the mandrel. The deformations of each level of helical structure (constructed by self-coiled and ultra-coiled) can be added, thus enhancing the contraction performance (see Fig. 2B).

The actuation principle of TUNA is based on the Joule heating generated by exciting the enameled copper wire, which causes radial expansion of the twisted nylon fibers. This expansion induces untwisting torque, which drives the helical structure to produce linear contraction. TUNA demonstrates a deformation of 60.2% under a load force of 0.5 N with an actuation stress of 0.44 MPa (0.3 N for loads, 0.2 N for slider). The gradual contraction process is illustrated in Fig. 2C and Movie S2. In addition, the ultra-coiled configuration can actively transition to the self-coiled state under higher load conditions, thereby preventing sudden breakage due to excessive weight. This structure is also reconfigurable, and under constrained spatial conditions, the self-coiled TNA can be folded medially to form a self-helix TNA, which further improves the output force of the TNA (see Fig. S7).

Notably, it is challenging to measure the temperature accurately and directly with an external sensor of TNAs. In our previous work [29,30], we proposed an integrated temperature self-sensing method based on the highly linear correlation between resistance and temperature of the enameled copper wire. This method utilizes the resistance of the enameled copper wire to calculate its temperature and further estimates the temperature of the TUNA (see Fig. S4). In this work, we conduct a detailed finite element analysis of heat conduction from the enameled copper wire to nylon fiber to determine its winding pitch and diameter. The FEM model of the TUNA is simplified as a cylindrical nylon fiber uniformly wound with copper wire. The initial temperatures of the nylon fiber and the copper wire are set to 25 °C, while the copper wire reaches 160 °C within 1.2 s and remains constant thereafter (see Fig. S2B). The thermal conductivities and convective heat transfer coefficients of the materials are listed in Table S1. The pitch of the copper wire varies from 0.4 to 1.0 mm, and its diameter is adjusted between 0.1 and 0.16 mm to investigate their effects on heat conduction. The temperature changes of the cross-sectional area of the nylon fiber under different conditions are obtained (the details are illustrated in Figs. S2 and S3 and Notes S2 and S3). The simulation results indicate that as the pitch increases, the distribution of the enameled copper wire becomes sparser within the same length, leading to a slower heat transfer rate due to reduced thermal contact area (see Fig. 2D). In contrast, the diameter has a limited effect on the temperature conduction rate. A wire diameter of 0.16 mm is selected to minimize the risk of breakage during the PTNA fabrication process. Consequently, the pitch and diameter of the copper wire are set to 0.4 and 0.16 mm, respectively.

In the nonactuated state, we demonstrate the passive load-stretch ratio characteristic of TUNA, which is as high as 396% (see Fig. 2E). TUNA can rapidly recover its original shape after releasing the load, demonstrating excellent energy storage capacity and adaptability to different loads. It can be observed that TUNA has a higher level of helical structure than TNA after being ultra-coiled. This results in more torsional insertions for the same length, allowing more excellent energy storage. To demonstrate energy storage capability, 5 TUNAs are fixed at one end and connected to a 3-dimensional (3D)-printed slider at the other, where a miniature basketball is placed (see Fig. 2F). After releasing the load (2.5 kg), the slider moves 20 cm within 0.05 s (the velocity reaches 4.0 m/s), and the basketball is thrown at approximately 131 cm in height (see Movie S6).

### Output force enhancement method based on a parallel-twisted structure

To enhance the output force of TNAs, we propose a parallel-twisted strategy to construct a stronger precursor fiber. In this approach, 4 pre-twisted nylon fibers are twisted in parallel and then self-coiled to fabricate a new type of TNA named parallel twisted nylon actuator (PTNA). Pre-twisting the fibers before paralleling facilitates the insertion of greater torsion number within the fiber bundle, thereby allowing for greater energy storage.

**Fig. 3.**
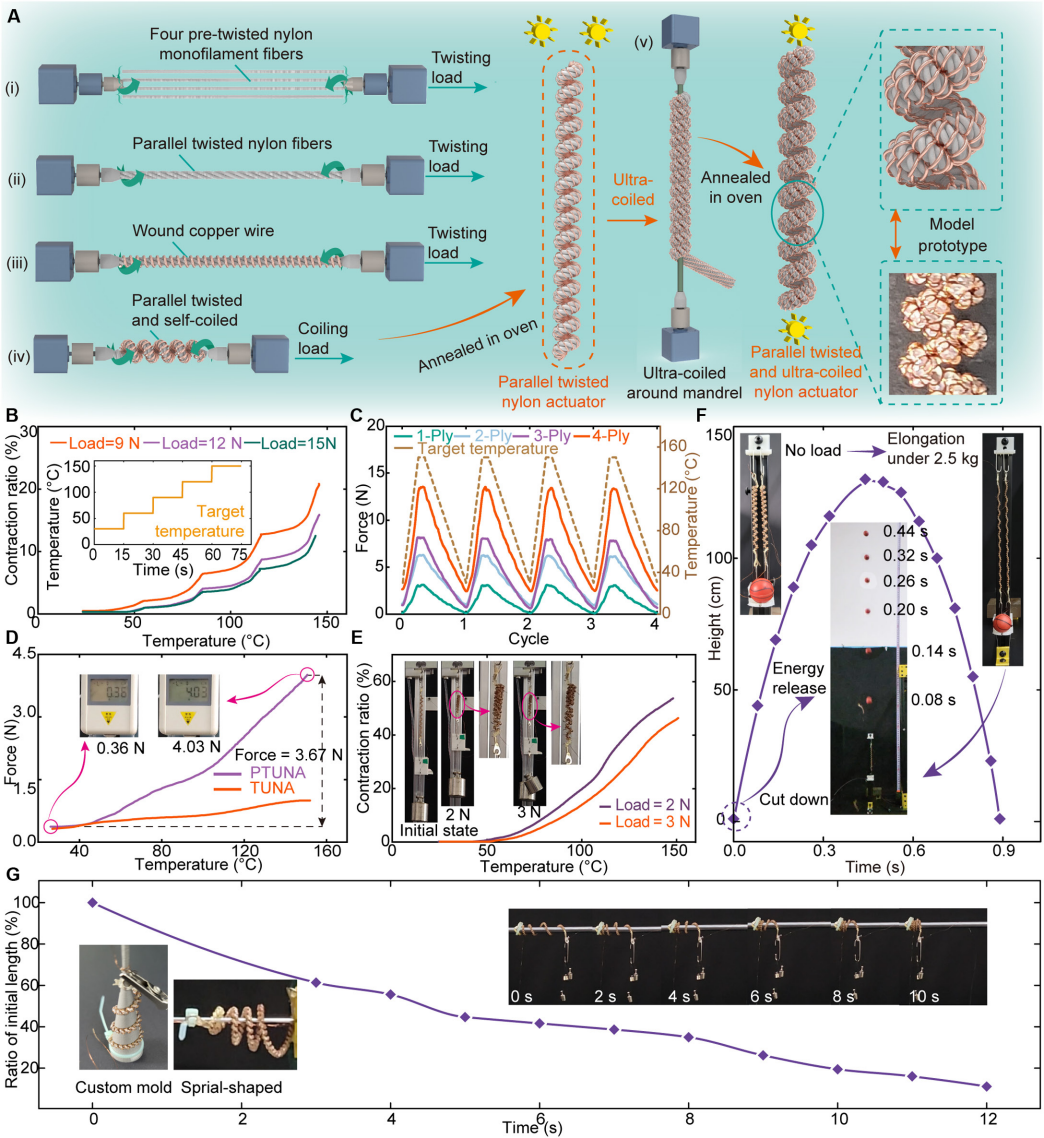
Design and experimental results of the PTNA and PTUNA. (A) Schematic of the fabrication process of the PTNA and PTUNA. (B) Contraction ratio of the PTNA under different load forces of 9, 12, and 15 N. (C) The output force of the PTNA with an increasing thread of fibers. (D) The output force of the PTUNA and TUNA. (E) Contraction ratio of the PTUNA under different load forces. (F) Energy storage capability of PTUNAs. (G) Spiral-shaped PTUNA achieves large deformation.

The detailed fabrication process of the PTNA is demonstrated [see steps (i) to (iv) in Fig. 3A and Movie S3]. First, 4 pre-twisted nylon fibers are arranged in parallel, and a stronger fiber with greater stiffness is constructed after preliminary twisting. The enameled copper wires are then uniformly wound around the stronger fiber, and continue twisting to form a stable self-coiled structure spontaneously. We further construct the dual-level helical structure based on the parallel-twisted method and fabricate another new type of TNA by ultra-coiled PTNA around a mandrel, which is named parallel twisted and ultra-coiled nylon actuator (PTUNA). PTUNA exhibits a higher load capability than TUNA due to the increase in stiffness of PTNA compared with TNA, thus providing enhanced output force (see Movie S5).

Initially, the relationship between the contraction ratio and the temperature of PTNA is tested. The target temperature is set to increase from 30 to 150 °C in increments of 30 °C every 15 s (see Fig. 3B). The experimental results indicate that PTNA can achieve a 20.3% deformation under a load of 9 N when heated to 150 °C, and a 12.1% deformation under a load of 15 N, while the actuation stress is 6.78 and 11.30 MPa, respectively (see Movie S4). We further compare the output force of PTNAs with varying numbers of fibers twisting in parallel (see Fig. 3C). The target temperature is set to 150 °C, increasing from room temperature within the first 0.25 cycle, remaining constant for 0.1 cycle, and then gradually decreasing back to room temperature. The experimental results show that a PTNA constructed from 4 nylon fibers can achieve an output force of 11.0 N (see Movie S4). Similarly, we test the output force of the PTUNA, applying a pre-tension force of 0.36 N. The display of the force sensor reaches 4.03 N, indicating that the output force reaches 3.67 N, which demonstrates a 439.7% improvement compared to the TUNA (see Fig. 3D). Additionally, the PTUNA can achieve contraction ratios of 53.7% and 46.3% under loads of 2 and 3 N, with actuation stresses of 1.51 and 2.26 MPa, respectively (see Fig. 3E). The energy storage capability of the PTUNA is measured and compared to the TUNA (see Fig. 3F). In the experiment, only 2 PTUNAs are selected, achieving similar results with 5 TUNAs, and the miniature basketball is thrown to 129 cm in height within 0.44 s (see Movie S6).

Moreover, the complete contact between adjacent coils in the helical structure limits the continued deformation and prevents further contraction. Accordingly, by emulating the configuration of a spiral spring, we place the self-coiled nylon fiber (TNA and PTNA) into a self-customized mold and fabricate the TUNA and PTUNA with a spiral-shaped ultra-coiled structure after annealing. The results indicate that the spiral-shaped PTUNA contracts over 88.9% under a load of 6 g in the horizontal direction (see Fig. 3G and Movie S5). Meanwhile, the spiral-shaped TUNA can achieve approximately 100% deformation (see Fig. S8 and Movie S2).

### Application of TNAs in bionic elbows and jumping robot

**Fig. 4.**
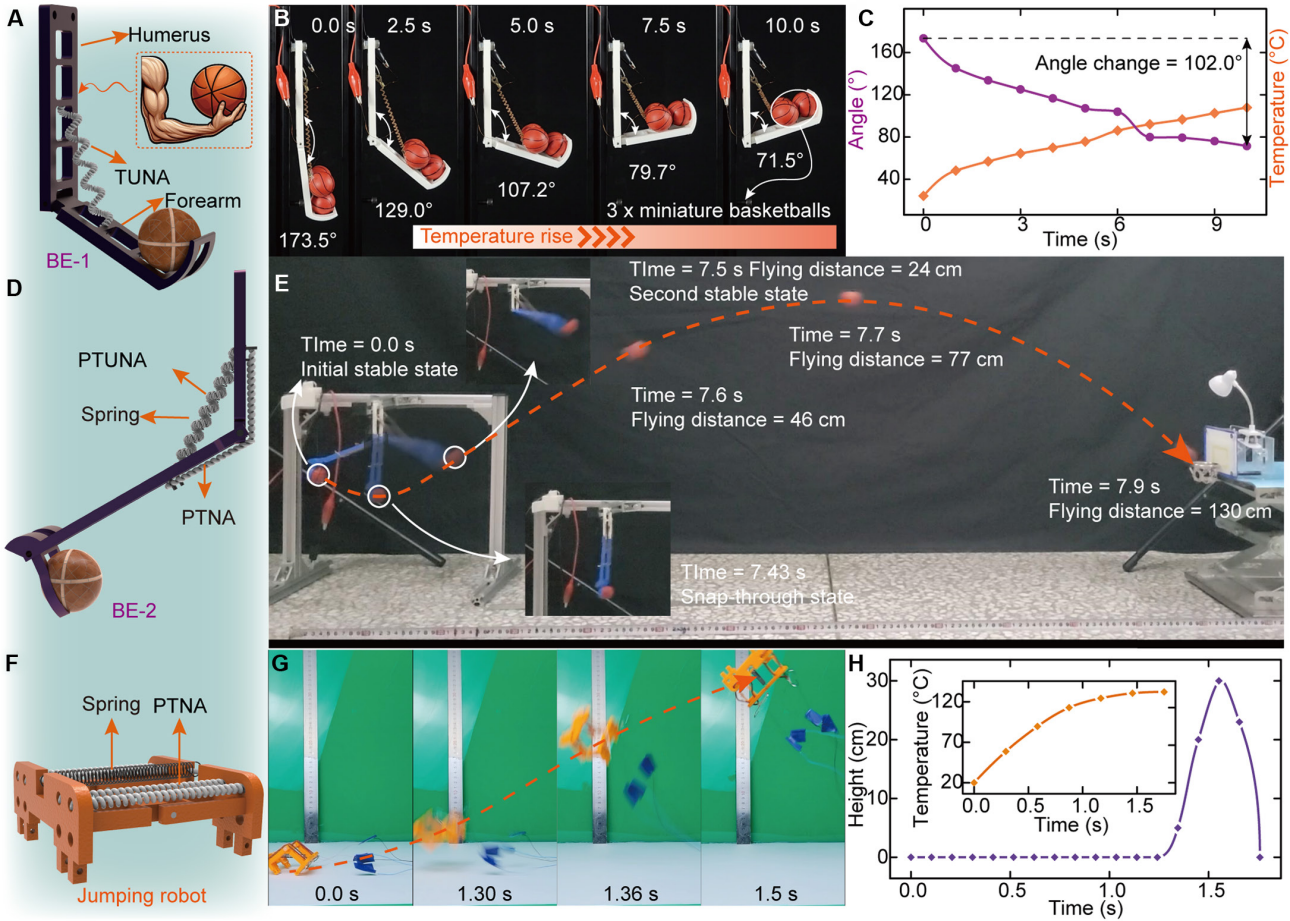
Soft robots can achieve lifting, rapid shooting, and jumping motion. (A) Configuration of BE-1 for lifting. It comprises rigid components resembling the humerus and forearm, with TUNAs affixed at the midpoint of the humerus and forearm, and the humerus is fixed to a bracket. (B) Lifting process from 173.5° to 71.5°. (C) The experimental results contain the time, angle, and temperature of the TUNA. (D) Configuration of the BE-2 for shooting. (E) Flying process of the miniature basketball. (F) Configuration of the jumping robot. (G) Jumping process within 1.5 s. (H) The experimental results contain the time, height of the robot, and temperature of the PTNA.

To validate the presented strategies and demonstrate the outstanding performance of 3 types of TNAs, we have adopted them to actuate some soft robots consisting of soft actuators and rigid components. We construct a scenario that mimics the actions of shooting basketball, which can be split into 2 stages: lifting and shooting the miniature basketball. These 2 stages correspond to the fundamental requirements for robots to perform similar actions. First, we develop a bionic elbow (named BE-1) driven by TUNA for lifting. Three miniature basketballs (22.5 g) are positioned at the base of the forearm (10 g), establishing an initial angle of approximately 173.5° between the forearm and the humerus (see Fig. 4A). We control the temperature of the TUNA to 110 °C within 10 s, and the elbow is actuated to rotate gradually (see Fig. 4B), achieving a rotation angle of 102.0°, showcasing the remarkable deformability of TUNA (Fig. 4C and Movie S7).

Next, the robot requires transient explosive force and deformation to shoot a miniature basketball, which is a challenge for TNAs. Therefore, we optimize the configuration of the bionic elbow by integrating energy-storing elements (spring) to create a bistable structure named BE-2 (see Fig. 4D). Capitalizing on the rapid transition between the bistable states facilitates the swift release of energy, enabling BE-2 to shoot the miniature basketball rapidly. We also establish a theoretical model to characterize the energy variation of BE-2, with detailed simulation presented in Fig. S9 and Note S6. The experimental results indicate that the BE-2 transitions from its initial stable state to the snap-through state, which occurs within the first 7.43 s. Within the next 0.07 s, it rapidly switches to the other stable state. The miniature basketball is released at 7.5 s and reaches a flight distance of 130 cm at 7.9 s (see Fig. 4E and Movie S7).

The bistable structure can rapidly release stored energy during the transition between 2 stable states, which is also applicable for propelling small robots to perform jumps and overcome higher obstacles. Ultimately, we propose a small jumping robot actuated by PTNA (see Fig. 4F) and analyze its energy conversion process. The theoretical calculations reveal that the greater the pre-stretch of the spring, the larger the difference between the maximum and minimum energy of the robot, which directly correlates with a higher jump height (see Fig. S10). The experimental results show that the robot can jump over 30 cm, which is 15 times its height (see Fig. 4G and H). It will aid in the application of small robots driven by PTNAs in exploratory work within unknown environments and even holds the potential to carry objects and surmount most obstacles.

### Application of TNAs in soft finger

**Fig. 5.**
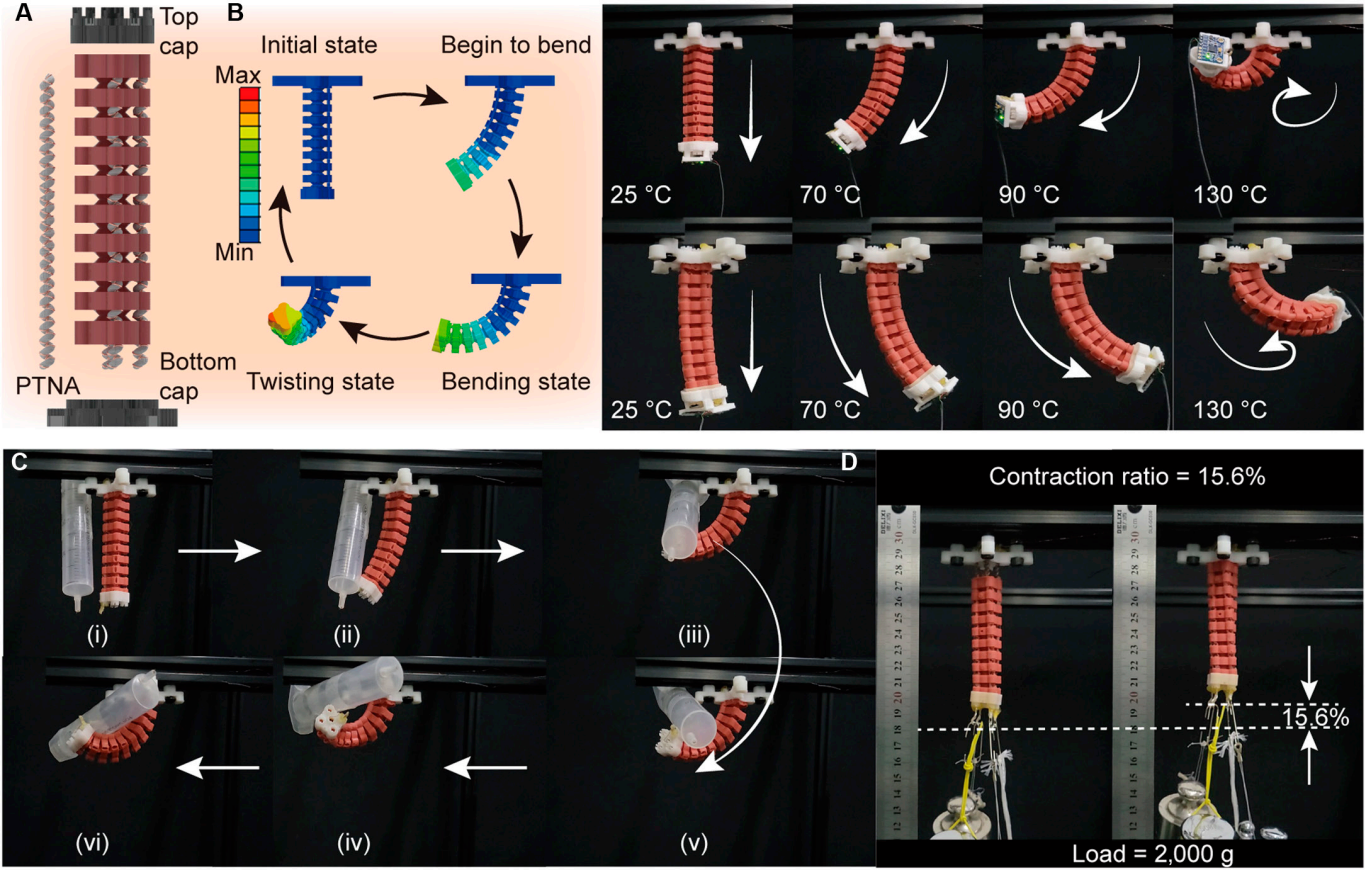
3-DOF soft finger enables contraction, bending, and twisting motions. (A) Schematic of the soft finger. (B) Bending and twisting motion driven by single and dual PTNAs. (C) Object-wrapped experiment. (D) Linear contraction experiments under a load of 2 kg.

By leveraging the inherent compliance of TNAs, we develop a 3-degree-of-freedom (DOF) soft finger driven by PTNAs, with a length of 80 mm and a radius of 10 mm, and the distance from the PTNA to the center is 7.5 mm (see Fig. 5A; the fabrication process is depicted in Fig. S11 and Note S7). It incorporates a notched cross-sectional design to augment the deformability, enabling it to perform bending, twisting, and contraction movements under substantial loads.

A finite element simulation of the soft module is performed to validate its deformation mechanism and the rationality of its geometric design. The bottom cap is defined as a rigid body and fully constrained, while the PTNA is coupled to the inner surface of the soft module. A temperature boundary condition is applied to the PTNA to induce contraction, and a moment boundary condition is imposed to simulate the twisting torque generated by PTNA during actuation. The results demonstrate that the soft module can produce a bending angle of 105° and a twisting angle of 96°, validating the actuation mechanism for multiple-mode motions.

Following the simulation validation, the deformation capability of the soft finger actuated by a single PTNA is further evaluated through experiments. The soft finger gradually bends along the orientation of the PTNA as its temperature increases. Once the notches in the bending direction are fully engaged, further bending is restricted. Instead, it begins to twist due to the torque exerted by the PTNA, and the torsional motion reaches approximately 90° when the temperature of PTNA reaches 130 °C (see Fig. 5B and Movie S8). The twisting motion of the soft finger is driven by the untwisting torque of PTNAs. Since this torque follows the opposite direction of the twisting process during fabrication, the twisting direction of the finger is opposite to that of the fiber during fabrication, which is clockwise. If a counterclockwise twisting motion is desired, the PTNAs should be fabricated with fibers twisted clockwise. Furthermore, we have verified the deformation process actuated by dual PTNAs, which is similar to that of a single PTNA actuation. Consequently, we progressively transition a cylinder object (syringe) with a diameter of 25 mm from its vertical orientation into a wrapped and enveloped state, as shown in Fig. 5C. This demonstrates the potential for capturing small objects. Next, the contractile capacity under various loads is evaluated (see Fig. 5D). The soft finger exhibits a 26.7% contraction ratio under no-load conditions and achieves 15.6% contraction deformation even under a load of 2 kg (see Movie S8).

**Fig. 6.**
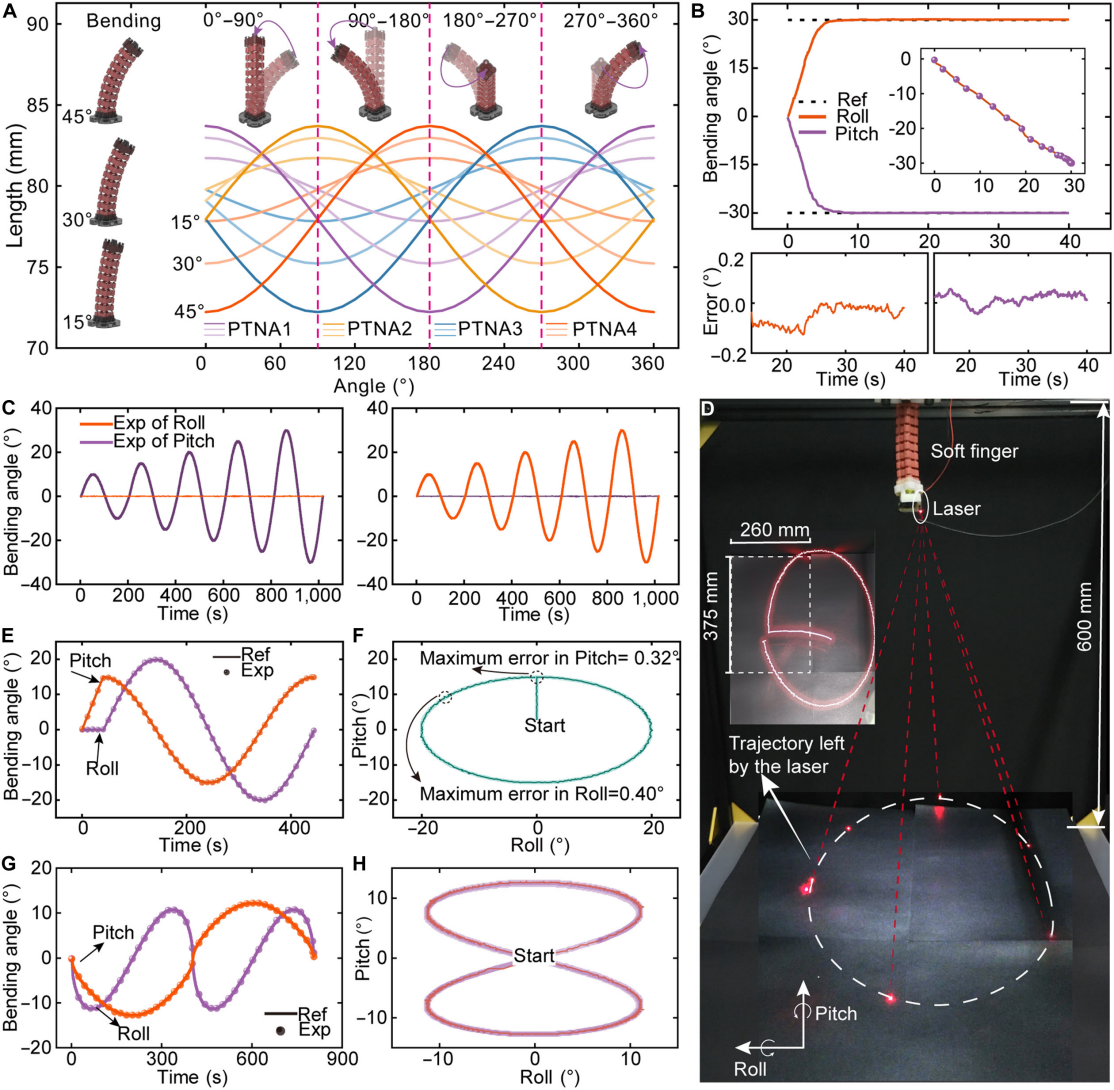
Attitude control experiments of the soft finger and applications in laser ablation. (A) Simulation results of length variations for 4 PTNAs during circumferential rotation of the soft module. (B) Point-control experiments of the bending angles and the errors in 2 directions. (C) Harmonic responses under increasing amplitudes. (D) Experimental setup for simulating the laser ablation. (E) Experimental results of bending angles for tracking the ellipse curve. (F) Trajectory for tracking the ellipse curve. (G) Experimental results of bending angles for tracking the “8”-like curve. (H) Trajectory for tracking the “8”-like curve.

The soft module demonstrates precise control over the bending and rotational motions, which is achieved through the coordinated actuation of the 4 PTNAs (Fig. S12). Based on the constant curvature model, we simulate the length variations of each PTNA during the circumferential rotation of the soft module, with initial bending angles of 15°, 30°, and 45° (see Fig. 6A). The simulation results indicate that a maximum length change of 7.8 mm is required for a single PTNA when the bending angle is 45°, and this calculation provides preliminary validation of the PTNA’s capability to achieve the required deformation. To further evaluate the module’s performance, a point-control experiment is conducted, setting the bending angles in both the Pitch and Roll directions to 30°. The rise time reaches 7 s, and the steady-state error is less than 0.13° (Fig. 6B). Subsequently, we test the attitude control performance following a sinusoidal trajectory with increasing amplitude in the Pitch and Roll directions (Fig. 6C). The maximum errors in the Pitch and Roll directions are 0.75° and 0.72°, respectively. When bending along the Pitch direction, the coupling of the Roll reaches 0.36°. Similarly, the coupling of the Pitch direction reaches 0.30°. The experimental results further validate the attitude control capability of the soft module (see Movie S9).

Finally, we integrate a miniature laser source at the terminal end of the soft finger to simulate laser ablation experiments (see Fig. 6D). The laser is set to track an elliptical path on the plane with amplitudes of 15° and 20°, and the path of laser is recorded. The temperature of PTNAs and tracking errors are illustrated in Fig. S13A and B. The variations in Pitch and Roll angles with the motion trajectories described by these angles are illustrated in Fig. 6E and F. The maximum tracking error in the Pitch direction occurs at 40.2 s. At this moment, the Pitch angle reaches its peak of 15°. In the Roll direction, the maximum tracking error is 0.40°, which occurs at 390.2 s. It can be attributed to the rapid tendency of the Roll angle to return to 0° (see Movie S10). Similarly, the bending angles and the trajectories when tracking a complex curve trajectory (like “8”) are demonstrated in Fig. 6G and H. The maximum error, which is also evident during the rapid turns, reaches 0.38° and 0.63° in Pitch and Roll angles, respectively (see Fig. S13C and D). The amplitude of the laser trajectory can be adjusted by varying the distance between the laser (mounted on the soft finger) and the target plane. This approach holds potential for future applications in the precise ablation of small biological tissues and various industrial laser processing domains.

## Discussion

In this work, we propose 3 strategies for TNAs to achieve large deformation, enhanced energy storage capability, and high output force, which are realized by constructing dual-level helical structures, parallel-twisted configurations, and their combination. The 3 novel types of TNAs (TUNA, PTNA, and PTUNA) validate the proposed strategies and are utilized to drive some representative soft robots. The bionic elbows achieve large amplitude and rapid movements, and the jumping robot jumps over 15 times the body height. Furthermore, the soft finger exhibits multiple-mode deformation involving contraction, bending, and twisting motions, as well as precise manipulation of the attitude.

To obtain large deformation and energy storage capability, we propose TUNA with dual-level helical structure. The initial helical structure is formed by the self-coiled process, and the second helical structure is constructed by the ultra-coiled process. The proposed TUNA offers several advantages: (a) Each level of the helical structure enables contraction, and the addition of the deformation from the 2 helical layers can amplify the overall deformation (60.2% of initial length). As a comparison, the TNAs made from nylon fibers are commonly fabricated through only self-coiled process in previous work. This method is straightforward to implement but is limited to achievable deformation. Besides, further twisting cannot be inserted into the nylon fiber after the self-coiled process due to the risk of breakage; thus, ultra-coiling around a mandrel is necessary. (b) The TUNA has an enhanced energy storage capability, allowing it to be elongated under load force in the nonactuation state, converting elastic potential energy into kinetic energy. It can quickly revert to its original length when the load force is suddenly removed, demonstrating the potential for use in projectile launching applications (the velocity of the slider reaches 4.0 m/s, and a miniature basketball is thrown to approximately 131 cm height, as shown in Movie S6). (c) The ultra-coiled configuration is reconfigurable and can transition into a self-coiled state under higher loads, preventing breakage from excessive weight. In constrained spaces, the self-coiled TNA can fold into a self-helix TNA, which effectively doubles its output force (Fig. S7).

To improve the output force of TNAs, we propose a parallel-twisted method to fabricate a stronger precursor fiber with higher stiffness. The fibers are pre-twisted to ensure much more torsion are inserted. For soft actuators, as well as rigid smart materials like piezoelectric stacks [38,39], a common method to enhance output force is through stacking actuators. Compared to stacked configurations [40,41], PTNA maintains a high output force (11.0 N) while exhibiting higher energy density and a more compact volume, making it more suitable for integrated applications. Meanwhile, we further construct the ultra-coiled structure utilizing the parallel-twisted method (based on PTNA) named PTUNA. The PTUNA further enhances the output force, achieving a 439.7% improvement and excellent contraction capabilities under higher loads than the TUNA, benefiting from the parallel-twisted method.

We comprehensively demonstrate the potential applications of the TUNA, PTNA, and PTUNA in soft robots. The developed robots exhibit substantial deformation, rapid actuation, multiple-mode deformation, and precise control. Specifically, leveraging the substantial contraction in TUNA, we propose a bionic elbow that achieves a larger rotation angle of 102.0°. Additionally, to address the slow actuation speed of TNAs, we integrate the bionic elbow with a bistable mechanism, enabling it to shoot a miniature basketball over 130 cm. This strategy is also used to design a robot that can jump over 30 cm, equipping it to explore unknown terrains. Finally, we develop a 3-DOF soft finger driven by PTNAs. Unlike the fiber-like artificial muscles like ​shape memory alloys (SMAs), which typically only contract linearly, TNAs can output twisting motions. The direction depends on the twisting direction during fabrication, enabling the finger to wrap around objects. Furthermore, the enhanced load capacity of PTNA allows the soft finger to achieve 15.6% contraction under a load of 2 kg. The precise control of the bending attitude is achievable through the coordinated operation of multiple PTNAs. In future work, TNAs could be further explored for wearable devices, offering enhanced motion range and sufficient force output. Additionally, they could be integrated into soft modules for biomedical applications like tissue ablation. However, since the TNAs proposed in this work are electrothermally actuated, their application is mainly constrained by the high operating temperatures and prolonged cooling times. Overcoming these limitations would greatly facilitate the practical implementation of TNAs.

## Materials and Methods

### Materials and fabrication of TNAs

The proposed TUNA, PTNA, and PTUNA consist of nylon 6,6 fiber and enameled copper wire, which were fabricated through self-customized equipment (Fig. S1). The equipment consists of 3 functional modules: (a) fiber twisted and self-coiled module; (b) electric heating wire winding module; and (c) ultra-coiled module. The diameter of the nylon fiber and copper wire was selected to be 0.5 and 0.16 mm, respectively.

### Control method of TNAs

TNAs were actuated by Joule heating generated by the enameled copper wire. The heat conduction process from the copper wire to nylon fiber was simulated by the finite element method (the details are provided in Figs. S2 and S3, Note S2, and Table S1). The simulation results allowed us to express the temperature of TNAs via that of the copper wire. Thus, a current and power monitor (INA226, Texas Instruments) was chosen to detect the voltage across the TNAs, which was subsequently converted into the resistance of the copper wire based on Ohm’s law. The temperature of TNAs could be estimated by leveraging the linear relationship between the temperature and resistance of the copper wire.

The temperature control system for TNAs was presented to achieve precise temperature control as follows. The current and power monitor was used to monitor the temperature; the MOSFET and power supply (S-120-12, Zhejiang Junlin Electric Technology Co., Ltd) were utilized to adjust the power; the MCU (STM32F103C8T6) was used to control the system.

### Testing platform for TNAs

A specialized testing platform was constructed to evaluate the characteristics of TNAs. It consists of the temperature control system, a resistive strain gauge force sensor, and a laser displacement sensor (BX-LV100N/R, Jingjiake Shenzhen). The displacement, force, and temperature data of TNAs were simultaneously controlled and plotted in real time on the computer (for detailed descriptions, see Fig. S5).

### Materials and fabrication process of the soft finger

The soft finger consisted of a silicone body, top and bottom caps, and 4 PTNAs. The main body was made of silicone (Ecoflex-0030, Smooth-On. Inc.), and the fabrication process is shown in Fig. S11. Two components of the mold were 3D printed, and 5 mandrels were inserted before pouring the mixed silicone. After injection, the top cover was closed, and the mold was placed in a vacuum degasser to eliminate air bubbles. Once the silicone was fully cured, the mold was opened to remove the completed soft finger. Four through-holes, each 3 mm in diameter, were evenly spaced around the body circumference to accommodate the PTNAs, with an additional central hole provided for wires. The 2 end caps were secured at both ends of the main body to hold the PTNAs in place.

## Data Availability

All data supporting the findings of this study are available in the paper and the Supplementary Materials.

## Supplementary Materials

Supplementary 1 Notes S1 to S7 Figs. S1 to S13 Table S1 Movies S1 to S10
