# Supplementary material for: High-Performance Twisted Nylon Actuators for Soft Robots
Source: Research (Wash D C). 2023 Mar 17;8:0642. doi: 10.34133/research.0642 (PMC11912876; doi:10.34133/research.0642)
Supplement: Supplementary 1 — Notes S1 to S7 Figs. S1 to S13 Table S1 Movies S1 to S10 [file research.0642.f1.zip › Supplementary Materials-R1-clean version.docx]

SUPPLEMENTARY MATERIALS

High-performance twisted nylon actuators for soft robots

Jin Sun†, Shijing Zhang†, Jie Deng, Jing Li, Dong Zhou, Dehong Wang, Junkao Liu, Weishan Chen, Yingxiang Liu*

State Key Laboratory of Robotics and Systems

Harbin Institute of Technology

Harbin 150001, China

*Corresponding author. E-mail: liuyingxiang868@hit.edu.cn

**This file includes:**

**Supplementary Notes**

Note S1. Fabrication equipment for TUNA, PTNA and PTUNA.

Note S2. Efficient actuating strategies for TNAs.

Note S3. Temperature control systems of TNAs.

Note S4. Development of a comprehensive testing platform.

Note S5. Characteristics of TUNA.

Note S6. Theoretical analysis of BE-2.

Note S7. Fabrication process and work principle of the soft finger.

**Supplementary** **Figures**

Figure S1. Self-customized equipment for fabricating the TUNAs, PTNAs, and PTUNAs.

Figure S2. Finite element simulation results of heat transfer process of TUNA.

Figure S3. Finite element simulation results of heat transfer process of PTNA.

Figure S4. Temperature self-sensing and control method of TUNAs.

Figure S5. Comprehensive testing platform for TNAs.

Figure S6. Output force testing platform.

Figure S7. Generation and elimination process of the ultra-coiled structure.

Figure S8. Deformation process of the spiral-shaped TUNA.

Figure S9. Design of the bionic elbow joint.

Figure S10. Design of the small jumping robot.

Figure S11. Fabrication process of the 3-DOF soft finger.

Figure S12. Typical states for the bending motion of the soft finger.

Figure S13. Experimental results of temperature and error for tracking different curves.

**Supplementary Table**

Table S1. Main Material Parameters of the TNAs.

**Other Supplementary Materials for this manuscript includes the following:**

**Supplementary Movies**

Movie S1. Fabrication and actuation process of TUNA.

Movie S2. Experimental results of TUNAs.

Movie S3. Fabrication and actuation process of PTNA.

Movie S4. Experimental results of PTNA.

Movie S5. Fabrication process and experimental result of PTUNA.

Movie S6. Energy storage capacity of TUNA and PTUNA.

Movie S7. Applications on small bionic robots.

Movie S8. 3-DOF soft finger.

Movie S9. Experiments on trajectory tracking of the soft finger.

Movie S10. Application experiments of the soft finger for wide-area laser ablation.

Supplementary Notes

**Note S1. Fabrication equipment for TUNA, PTNA and PTUNA**

We have developed self-customized equipment to fabricate three types of TNAs (Fig. S1A). The fabrication process of TNAs requires several steps, including nylon fiber twisted, electric heating wire wind, self-coiled, and ultra-coiled around the mandrel.

Therefore, the equipment includes the following functional modules: 1) Fiber twisting and self-coiling module: This module is used to twist the nylon fiber and achieve a self-coiled structure. It includes two stepper motors, motor-1 and motor-2, which rotate opposite directions to drive the nylon fiber to twist clockwise or counterclockwise. The nylon fiber is fixed between the two motors, with one motor fixed and the other capable of axial movement. The counterweight is attached to the end to apply continuous tension and prevent fiber instability. 2) Electric heating wire winding module: This part consists of a motor-lead screw system and a custom slider. During operation, the heating wire passes through designated holes in a specific device, then motor-1 and motor-2 rotate in the same direction while motor-3 drives the uniform movement of the heating wire. This process uniformly winds the enameled copper wire onto the twisted nylon fiber, ensuring efficient heat transfer from the heating wire to the nylon fiber. The winding pitch can be adjusted by the ratio of the motor’s movement speed to its rotation speed. 3) Ultra-coiled module: This module includes motor-4, motor-5, a bearing, and a screw slider. During operation, the mandrel is fixed between motor-4 and the bearing. One end of the self-coiled nylon fiber is attached to the mandrel, and the other is connected to a load to apply pre-tension. Motor-4 rotates in coordination with the movement of the slider, uniformly winding the self-coiled nylon fiber around the mandrel to form an ultra-coiled structure, a key step in fabricating TUNA and PTUNA.

The fabrication processes of TUNA and PTUNA require the coordinated operation of three modules, as shown in Fig. S1B. PTNA requires the twisting module and the electric heating wire winding module to work. Besides, the twisting module is also used for parallel twisted fibers to achieve a twisted and self-coiled process.

During fabrication, annealing under twisted states is an effective method for locking the helical structure of TNAs. Haines et al. investigate the effects of annealing on twisted fibers (see reference [1] in the main text), and provide valuable insights into how thermal treatment influences polymer chain reorganization, which can be discussed from two perspectives:

(a) Thermal expansion behavior:

Haines et al. reported that non-twisted fibers contract by 2.5% when heated between 20°C and 180°C, while twisted fibers exhibit a reduced contraction of 1.7% due to twist-induced helical rearrangement of polymer chains. Experimental results indicate that annealing further reduces axial contraction in twisted fibers, suggesting that axially oriented molecular chains undergo structural reconfiguration into a helical conformation, which restricts free axial contraction and enhancing structural stability.

(b) Glass transition temperature (*Tg*):

According to their findings, the *Tg* of non-twisted nylon fibers is 70°C, which increases to 89°C when fully coiled after annealing. In their experiments, a 19°C increase in *Tg* was observed under twisted conditions, indicating that the combined effect of twisting and annealing enhances polymer chain orientation stability. This stabilization may also facilitate the transition of amorphous chains into more ordered structures, thereby improving the mechanical stability of TNAs at elevated temperatures.

## Note. S2 Efficient actuating strategies for TNAs.

TNAs (including TUNA, PTNA, and PTUNA) are thermally driven soft actuators composed of nylon fibers and electric heating wire (we select enameled copper wire here). The deformation of TNAs is achieved by the Joule heating generated by current through the enameled copper wire. The method of combining the enameled copper wire and nylon fibers directly determines the efficiency of the temperature rise of the nylon fibers, thus affecting the actuation performance.

We simplify the structure of the TUNA as illustrated in Fig. 1, and the efficient driving method for TNAs is transformed into the problem of choosing the appropriate diameter of the enameled copper wire and its winding pitch on the nylon fiber. Thus, we establish a finite element model of the copper wire uniformly wound on the nylon fiber (Fig. S2A), with the properties of the materials shown in Table S1. A thermal condition is applied to the copper wire (Fig. S2B), and the temperature change of the cross-sectional area of the nylon fiber with time can be obtained.

We designed two simulations to investigate the influence of winding pitch and diameter of the enameled copper wire, separately. Firstly, we choose the diameter of the enameled copper wire to be 0.1 mm. The winding pitch of the copper wire on the nylon fiber is controlled to be 0.4 mm, 0.6 mm, 0.8 mm, and 1.0 mm, respectively. The temperature change of the central cross-section of the nylon fiber and the minimum, maximum, and average temperatures are shown in Figs. S2C to S2F, respectively. As the pitch increases, the distribution of the enameled copper wire becomes sparser within the same length, the heat transfer speed slows down, and the time for the center temperature to reach a steady state gradually increases from 1.5 s to about 4 s. Therefore, we choose to wind the enameled copper wire with a pitch of 0.4 mm.

Then, the pitch is set to 0.4mm, and the diameter of the enameled copper wire is set from 0.1mm to 0.18mm. We explore the change in the center temperature of the nylon fiber under different conditions. The simulation results show that the diameter of the electric heating wire has a negligible impact on the rate of temperature conduction. Besides, in the fabrication process of PTNA, the self-coiled process subjects the enameled copper wire to shearing forces. Therefore, to minimize the risk of wire breakage, the diameter of the enameled copper wire is set to 0.16 mm for further research.

For the PTNA and PTUNA, it is necessary to analyze the influence of the parallel number of nylon fibers during the heat transfer process. Based on the above simulation conditions, we simplify the parallel fibers of different numbers into nylon fibers with increasing diameters. The simulation of the heat conduction speed from the electric heating wire to the center of the nylon fiber under the parallel conditions of 2, 3, 4, and 5 nylon fibers (described as 2-5 ply) is implemented. The results show that the heat conduction speed slows as the number of fibers increases (Fig. S3). The average temperature of 5-ply PTNA at 2.0 s decreases to 138.3°C. Therefore, we choose 4-ply fibers for parallel twisting and winding.

Additionally, we have evaluated the correlation between applied voltage and contraction rate for the three types of TNAs. In the experiments, the applied voltage ranged from 4V to 8V with a current limit of 3A, and a DC power supply is used for actuation. Among the three types of TNAs, TUNA exhibits the fastest deformation rate, achieving a displacement of 53.5 mm within 2.5 s at 8V, with a corresponding contraction rate of 21.4 mm/s. PTNA exhibits the slowest deformation rate due to its lower contraction ratio compared to the other two TNAs. However, it is more suitable for applications requiring higher output force. PTNA deforms 30.6 mm within 6 s at 8V, resulting in a contraction rate of 5.1 mm/s.

Moreover, we use PTNA as an example to estimate the energy conversion efficiency of TNAs, which can be defined as the ratio of mechanical energy output to the thermal energy input required during the actuation process. For PTNA, it can lift a load of 1.5 Kg by 12.0 mm, the mechanical energy output during contraction is 0.177J. During actuation, the temperature of the copper wire increased from 25℃ to 150℃, and the thermal energy consumption can be estimated as 7.22 J. The energy conversion efficiency is approximately 2.45%. As electrothermal actuators, TNAs exhibit limited energy conversion efficiency, which remains a constraint on their practical applications. Therefore, developing alternative actuation mechanisms, such as magnetically or fluid-driven TNAs, represents a promising research direction.

**Note. S3 Temperature control systems of TNAs.**

The enameled copper wire and nylon fiber are close and in sufficient contact with each other. The FEM simulation results indicated that the average temperature of the nylon fiber and copper wire essentially reaches the same within 2 s. Thus, we assume that the temperature of the enameled copper wire is essentially the same as the temperature of the TNAs, and use it to represent and control the temperature of the TNAs (TUNA, PTNA, and PTUNA). Leveraging the linear relationship between the temperature and resistance of the enameled copper wire, we can estimate the temperature of the TNAs by measuring the real-time resistance of the enameled copper wire. Specifically, a current and power monitor (INA226, Texas Instruments) is selected to obtain the resistance by detecting the voltage and current, the principle is shown in Fig. S4A. The voltages across the sampling resistor and the TNAs are alternately measured. Since the sampling resistor is in series with the actuator, Ohm's law allows us to convert the measured voltage and current into the resistance of the enameled copper wire in the TNA and subsequently estimate the temperature of TNA. This enables intrinsic temperature self-sensing without external sensors.

Furthermore, we developed a temperature control system for TNAs. The system utilizes the INA226 chip to acquire the temperature of TNAs as mentioned before. A MOSFET is employed to adjust the output voltage of a power supply (S-120-12, Zhejiang Junlin Electric Technology Co., Ltd), thereby controlling the input power of TNAs and achieving precise temperature control. The hardware diagram of the system is shown in Fig. S4B. The software component is a control program based on an MCU (STM32F103C8T6), as illustrated in Fig. S4C. It can realize Bluetooth communication, data acquisition and feedback, instruction reception and parsing, temperature control, and drivers for additional peripherals.

**Note. S4 Development of a comprehensive testing platform.**

To automate the testing of various performance characteristics of different TNAs, we develop a comprehensive testing platform, as shown in Fig. S5. The testing platform comprises the temperature control system with its control board, a resistive strain gauge force sensor, a laser displacement sensor (BX-LV100N/R, Jingjiake Shenzhen), and a torque motor (70TP06DV22, Jscc Automation LTD.). In the experimental process, one end of the TNA is fixed on the force sensor, and the other is connected to a slider. The slider can either suspend weights directly or connect to a torque motor via a capstan, thereby applying constant or periodically varying loads to the TNAs. The displacement, force, and temperature data of the TNAs are simultaneously collected and controlled by these sensors.

Additionally, the output force of the PTNA is measured by a professional force gauge (HP-50, Yueqing Handpi Instruments Co., Ltd.), which is fixed on an adjustable stand. One end of the PTNA is connected to the force gauge, and the other is secured with a machine vise, and the pre-tension force of the PTNA is controlled by the adjustable stand before the experiment. The relationship between the output force and the temperature of the PTNA can be obtained (Fig. S6).

**Note. S5 Characteristics of TUNA.**

TUNA consists of a dual-level helical structure that is constructed by ultra-coiled TNA around a mandrel. The inner helical structure is formed by self-coiled nylon fibers, and the external helical structure results from the further ultra-coiling around the mandrel. The deformation of each level of helical structure (constructed by self-coiled and ultra-coiled) can be added, thus enhancing the contraction performance (Fig. 2B). The parameterization equation of TUNA can be expressed as:

where *R*1, *R*2, and *p*1, *p*2 denote the radius and pitch of the ultra-coiled and self-coiled helical structures, respectively. Besides, *φ* is the parameter used to characterize the first level helical structure. *θ* describes the position along the secondary helical structure that spirals around the first helix, it controls the rotation around the current position on the first helix.

It's worth noting that the ultra-coiled structure of the TUNA can be controllably eliminated and regenerated (Fig. S7). The TUNA does not contract when heated under significant load force; instead, it elongates as the ultra-coiled structure gradually dissipates, reverting to a typical TNA that can be actuated. Following the steps shown in Fig. 2A, the TNA can be re-ultracoiled around a mandrel to reconstruct the TUNA.

Additionally, in specific applications where actuator length is strictly limited and greater output force is required, we can forgo reconstruct the ultra-coiled configuration and fold the reverted TNA in the middle to create a self-helix TNA, which was proposed in our previous research utilizes the torsional moment of the TNAs. It can lead adjacent TNAs to twist around each other during deformation, significantly enhancing the output force.

**Note. S6 Theoretical analysis of BE-2.**

The proposed BE-1 driven by TUNA can rotate significantly to carry three miniature basketballs but cannot achieve a fast-shooting motion. We present a combination design of a bistable mechanism with BE-1 to construct BE-2, which leverages the rapid motion characteristic of the bistable mechanism to achieve fast shooting. BE-2 consists of an energy storage element (spring) driven by PTNA and PTUNA, as shown in Fig. S9A. The PTNA is gradually heated from the initial state, and the PTUNA is activated when BE -2 reaches an unstable state (snap-through state). Then, it quickly reaches another stable position to shoot the miniature basketball.

In the initial state, the spring is relaxed, and the PTNA is stretched. BE-2 slowly swings to the intermediate stable position with the PTNA beginning actuation. Subsequently, the PTUNA is heated and rapidly swings BE-2 to the other stable position, converting the stored elastic potential energy into kinetic energy, thus enabling a quick swing that shoots the miniature basketball.

The elastic potential energy of the system can be expressed as:

where *UTotal*, *USpring*, and *UPTNA* denote the elastic potential energy of the system, the spring, and PTNA, respectively. Specifically, the elastic potential energy spring can be represented as:

where *kS* is the stiffness of the spring, *∆lS* is the length change of the spring, which can be calculated as:

where *lpre* is the pre-extension length of the spring, *l*1 and *l2* denote the effective length of the spring from the fixation to the center pivot, respectively, and *θ* expresses the angle between the steady state and the snap-through state.

The elastic potential energy spring can be calculated as:

Consequently, the change in elastic potential energy of the system can be represented as:

Similar analysis results for the jumping robot are illustrated in Fig. S10.

Then, we discuss the relationship between the shooting displacement and the initial angle (Fig. S9B). The velocity of the miniature basketball before and after shooting can be described as follows:

where *v0* is the initial velocity and *v1* is the terminal velocity. *α* and *β* denote the angles between the initial and terminal velocities and the horizontal direction, respectively.

The displacement of the miniature basketball can be calculated as:

The condition for the displacement to obtain a maximum value is .

Furthermore, as shown in Fig. S9C, the geometrical relation leads to:

where *h* expressed the difference in height between the throwing and landing points of the miniature basketball, and the equation can be expanded as:

The condition for the equation to have a solution is:

The result indicates that when *h* is close to zero, we can set *α* to 45° to get the maximum displacement.

**Note. S7 Fabrication process and work principle of the soft finger.**

The soft finger comprises a silicone body, top and bottom caps, and four PTNAs, and the fabrication process is shown in Fig. S11. The mold comprised two 3D-printed components, into which five mandrels were inserted prior to pouring the prepared silicone mixture. Following injection, the top cover was sealed, and the mold was placed in a vacuum degasser to remove any entrapped air bubbles. After the silicone was fully cured, the mold was carefully opened to release the complete soft finger.

The PTNAs are positioned a certain distance from the center axis of the soft finger, and a single PTNA could drive the soft finger to achieve bending and twisting motions. Additionally, the soft finger will contract when all four PTNAs are heated simultaneously. Each PTNA is connected to an independent temperature self-sensing control circuit, allowing for individual closed-loop temperature control. With the coordinated excitation of the four PTNAs, the soft finger can be driven to bend and sweep in different directions. The bending attitude can be precisely controlled based on the temperature control of PTNAs.

A sectional view of the soft finger is illustrated in Fig. S12, and the PTNAs are labeled as PTNA-1 through PTNA-4. There are six typical states for the bending motion of the end of the soft finger. As shown in State-1 and State-4, when a single PTNA-1 is excited, the soft-body finger bends toward the PTNA axis. When two adjacent PTNAs are actuated simultaneously, the soft finger is deflected between two axes.


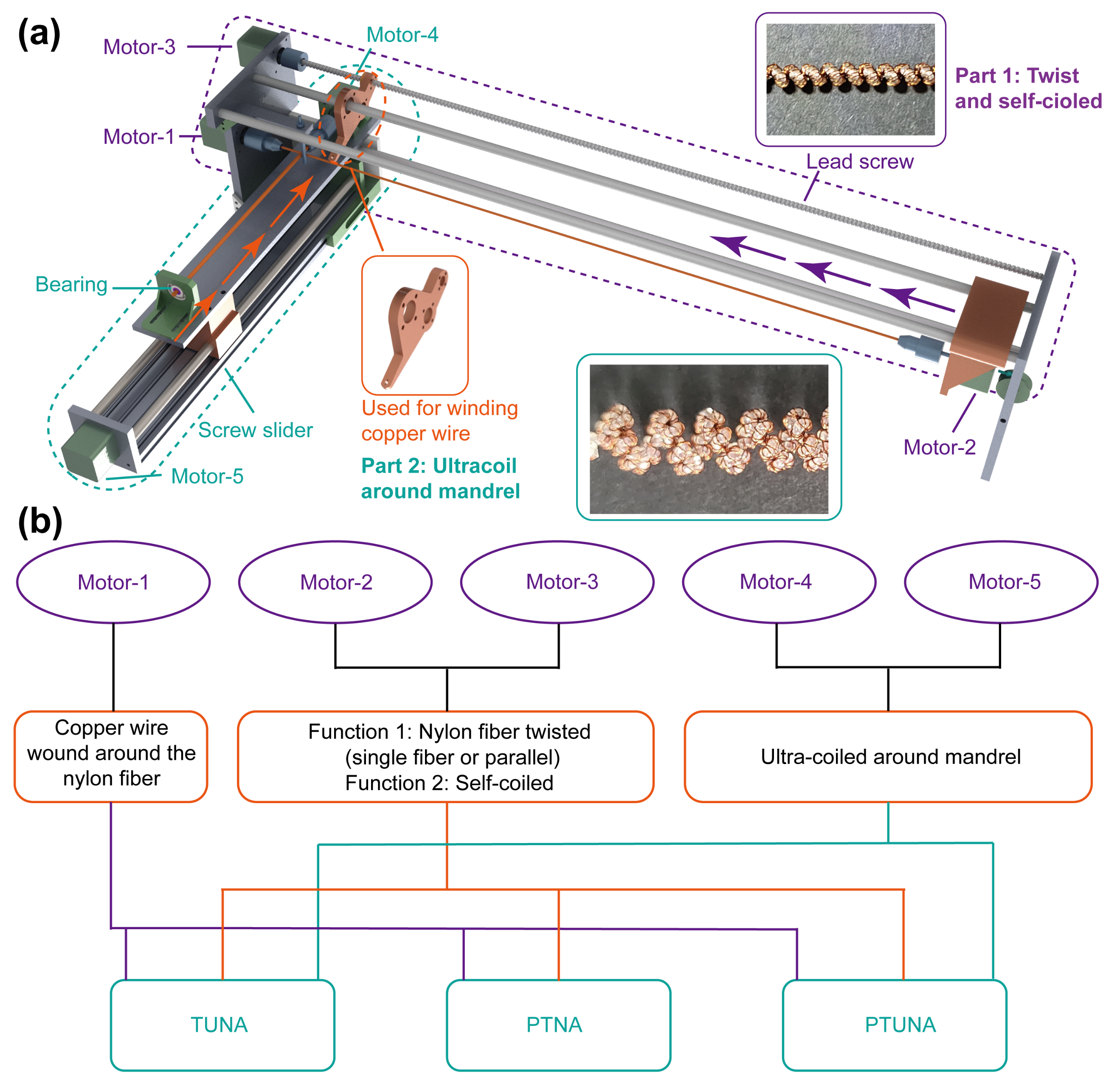


Fig. S1. Self-customized equipment for fabricating the TUNAs, PTNAs, and PTUNAs. (A) Model of the equipment. (B) Schematic from motors to functions and three types of TNAs.

**
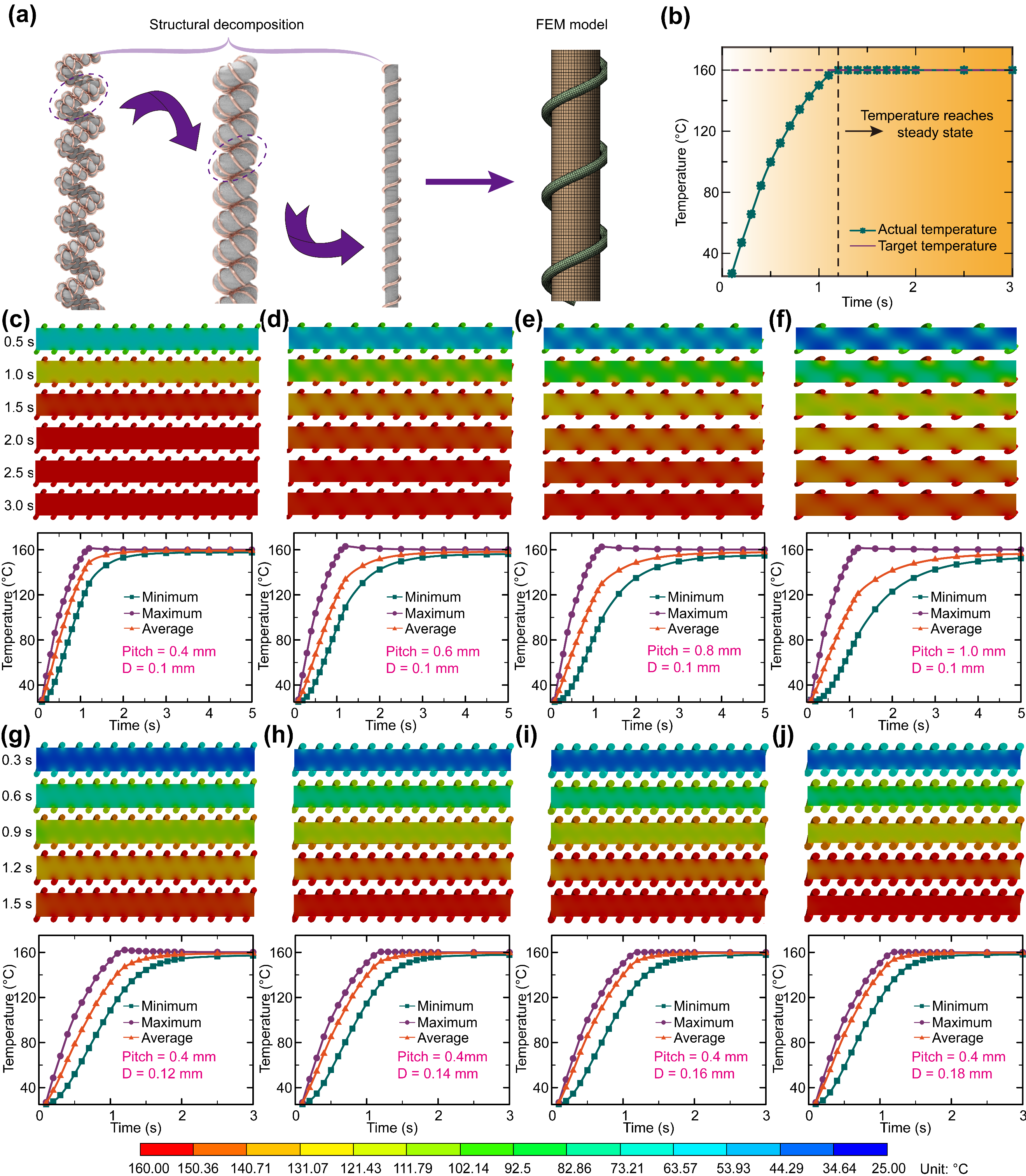
**

Fig. S2. Finite element simulation results of heat transfer process of TUNA. (A) Finite element model obtained by structural simplification. (B) Input temperature of enameled copper wire. (C-F) Temperature variation of TUNA for enameled copper wire winding pitch of 0.4mm, 0.6mm, 0.8mm and 1.0mm, respectively. (G-J) Temperature variation of TUNA for enameled copper wires with diameters of 0.12mm, 0.14mm, 0.16mm and 0.18mm, respectively.

**
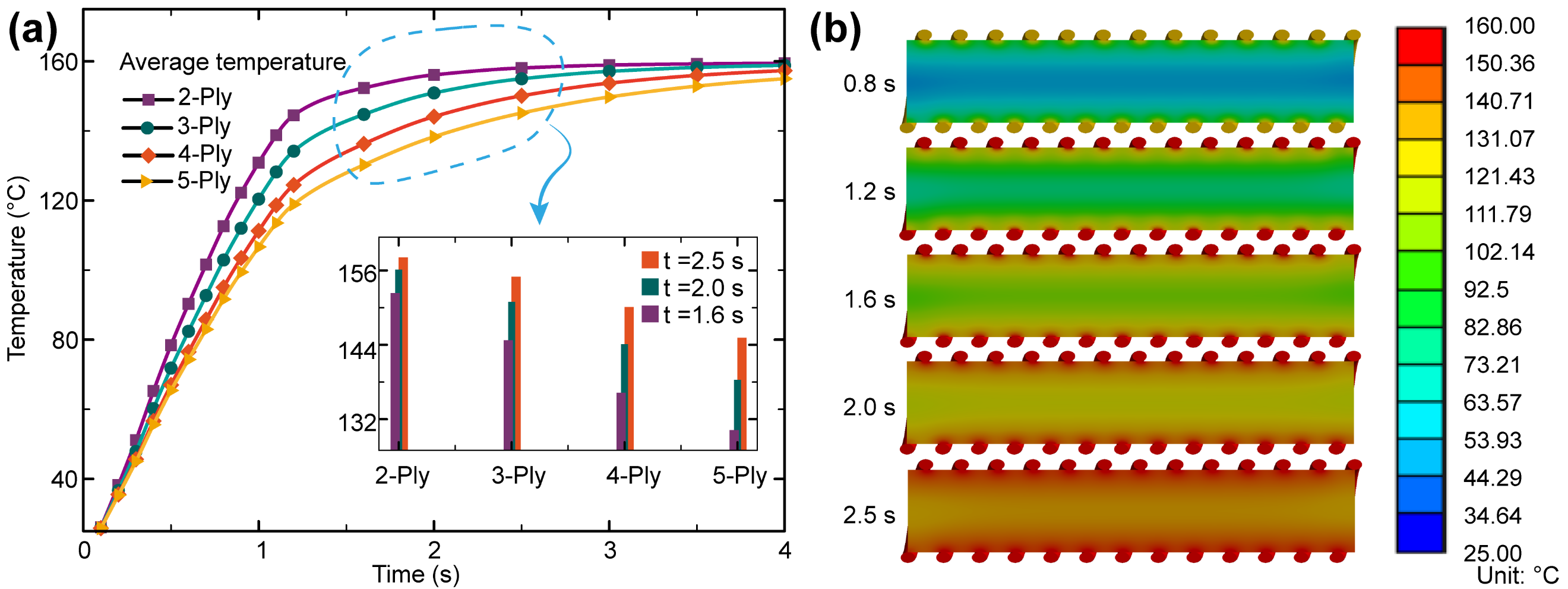
**

Fig. S3. Finite element simulation results of heat transfer process of PTNA. (A) Temperature change of PTNA made of different numbers of nylon fibers. (B) Heat transfer cloud diagram of the 4-ply PTNA.


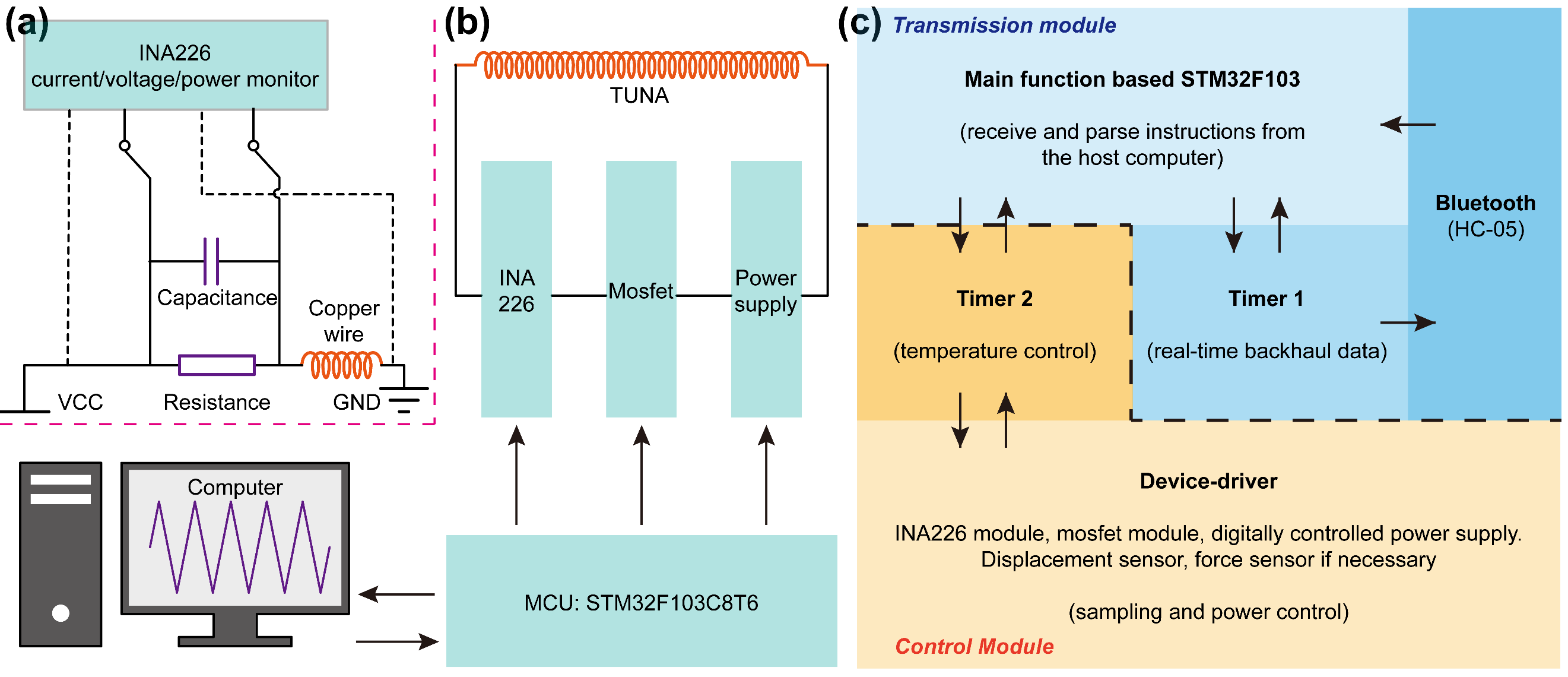


Fig. S4. Temperature self-sensing and control method of TUNAs. (A) Measuring principle of the INA226. (B) Diagram of the hardware structure of the control system. (C) Diagram of the software structure.


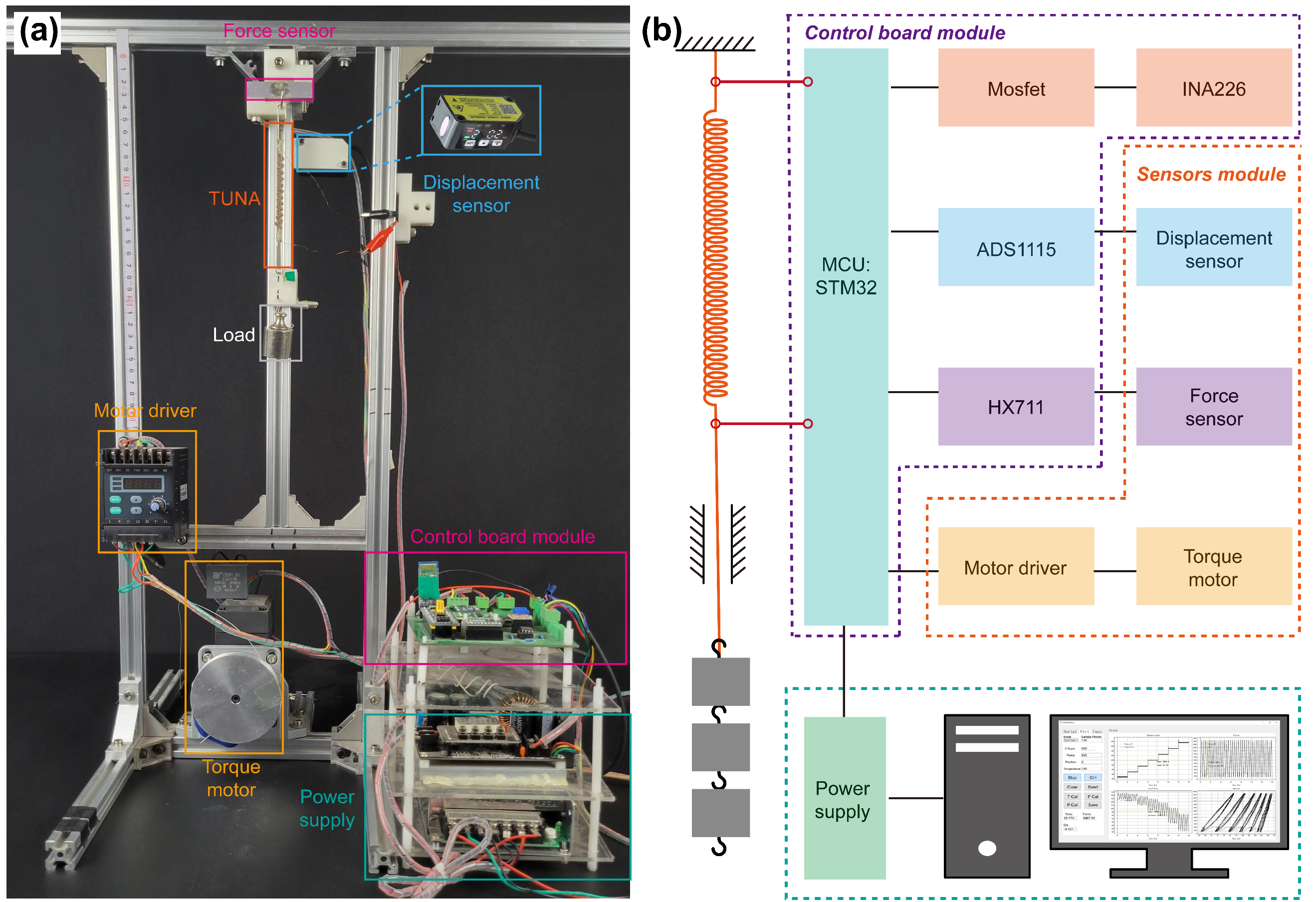


Fig. S5. Comprehensive testing platform for TNAs. (A) Front view of the platform. (B) Diagram of the hardware structure.


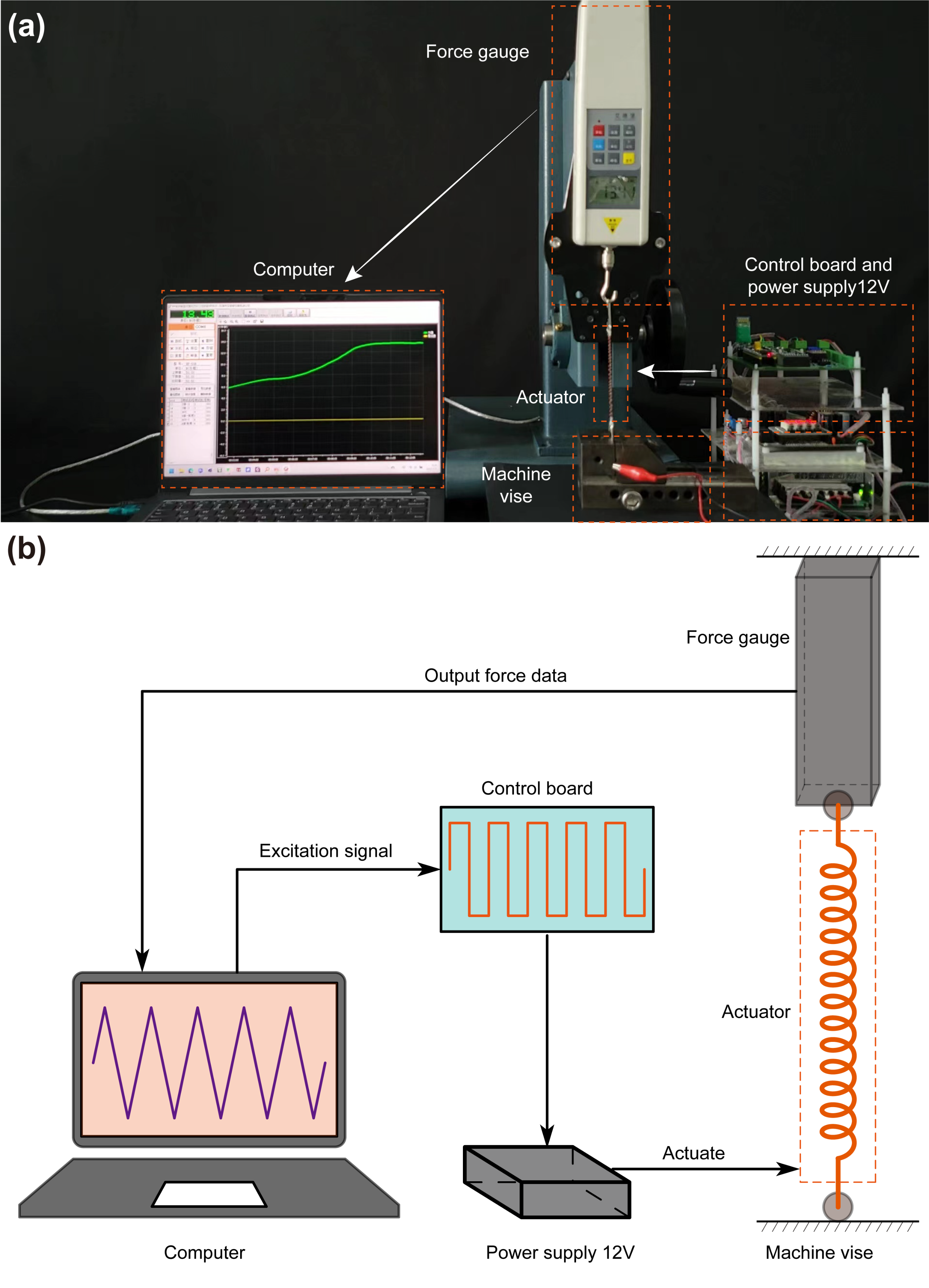


Fig. S6. Output force testing platform. (A) Front view of the platform. (B) Diagram of the platform.


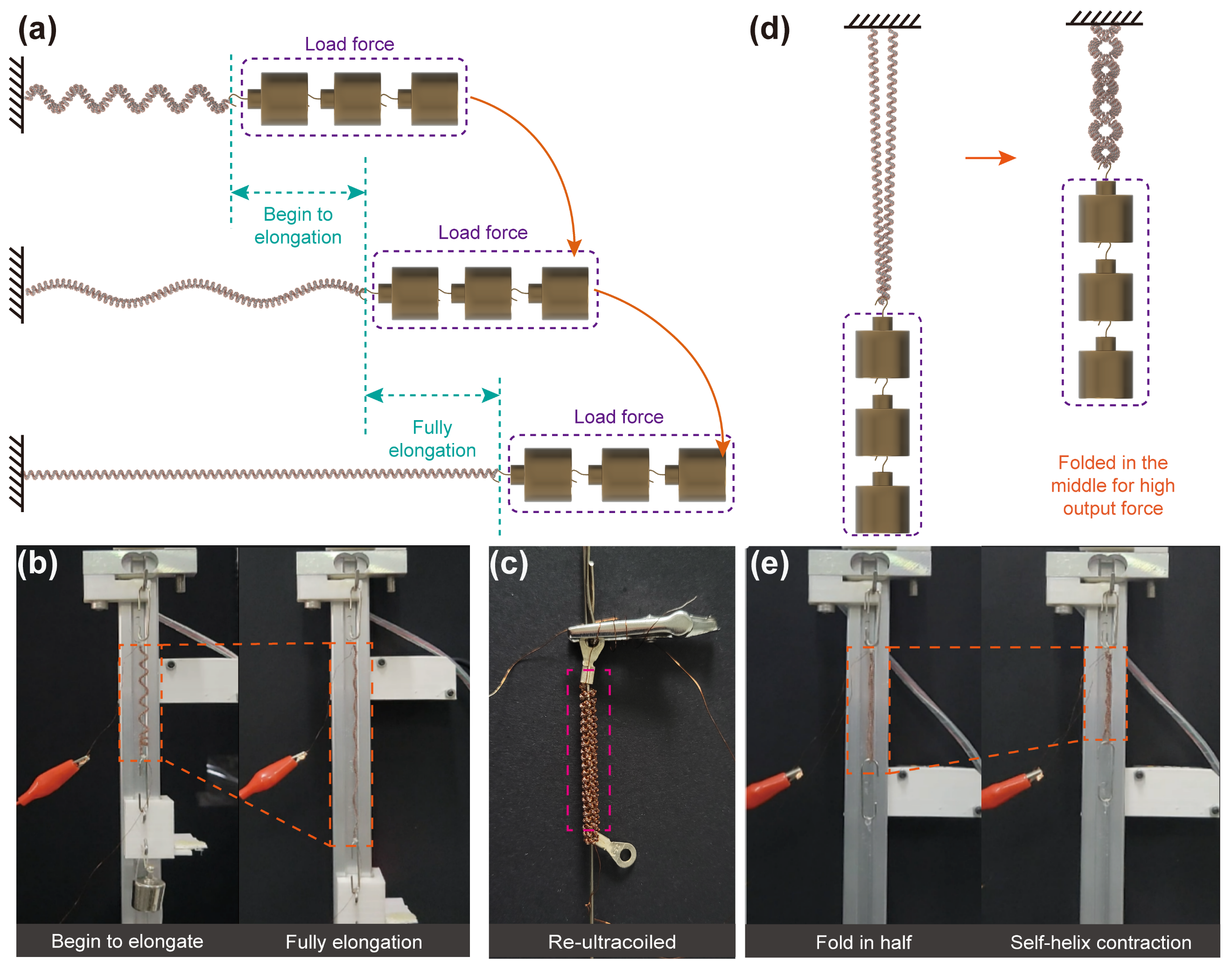


Fig. S7. Generation and elimination process of the ultra-coiled structure. (A-B) Elimination process from the TUNA to TNA. (C) TNA can be re-ultra-coiled to TUNA. (D-E) TNA can be folded in half to construct the self-helix TNA for higher output force.


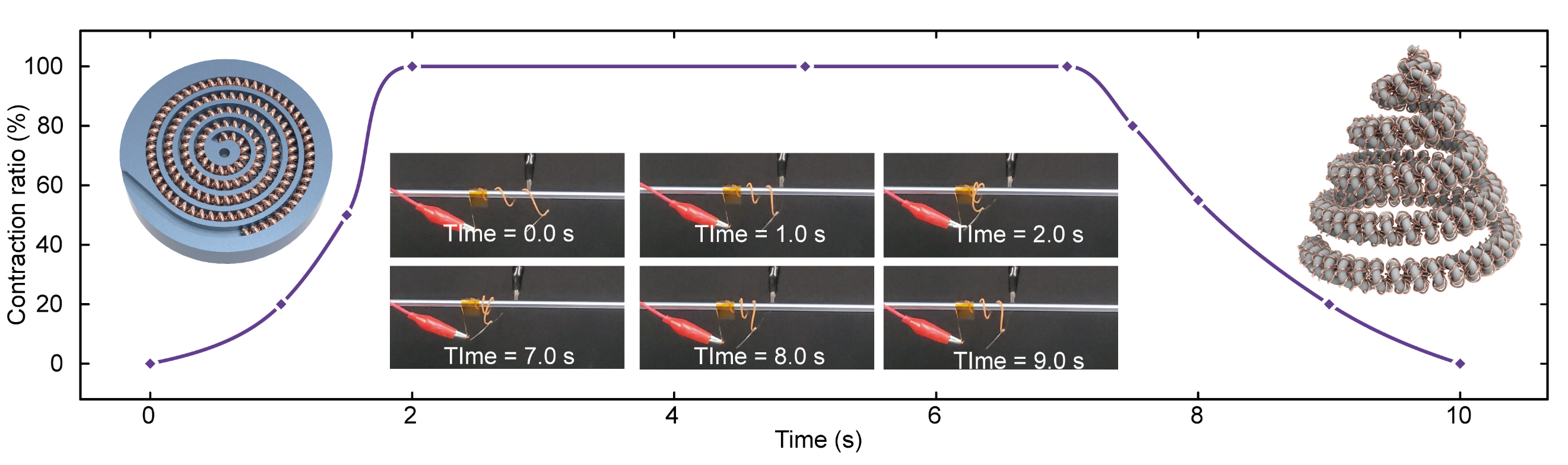


Fig. S8. Deformation process of the spiral-shaped TUNA.


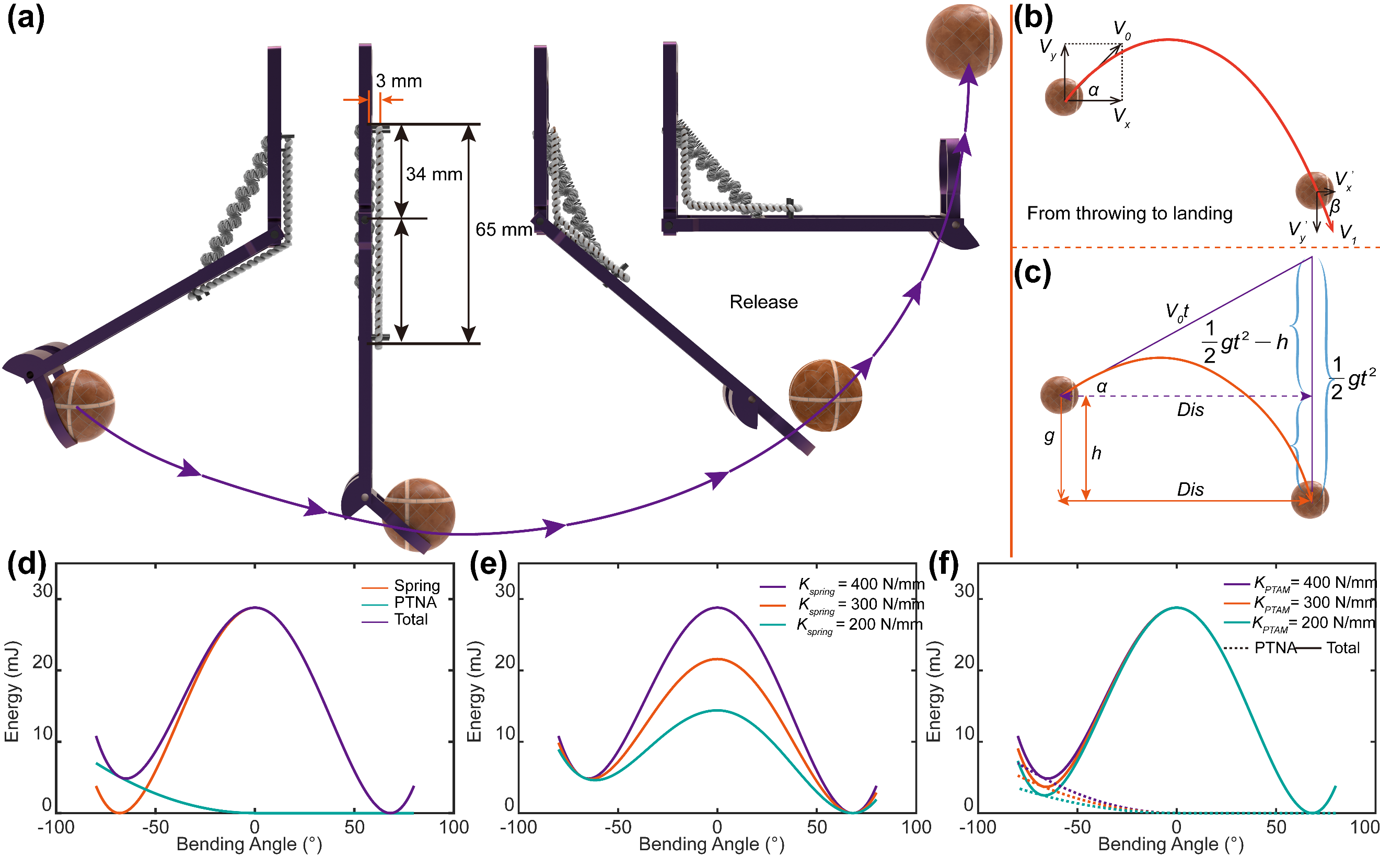


Fig. S9. Design of the bionic elbow joint. (A) Diagram of shooting a miniature basketball. (B) Analysis of the angle of emission. (C) Analysis of shooting height and distance. (D) Energy changes during motion. (E) Relationship between the energy change of the system and the stiffness of the spring. (F) Relationship between energy changes and stiffness of PTNAs.


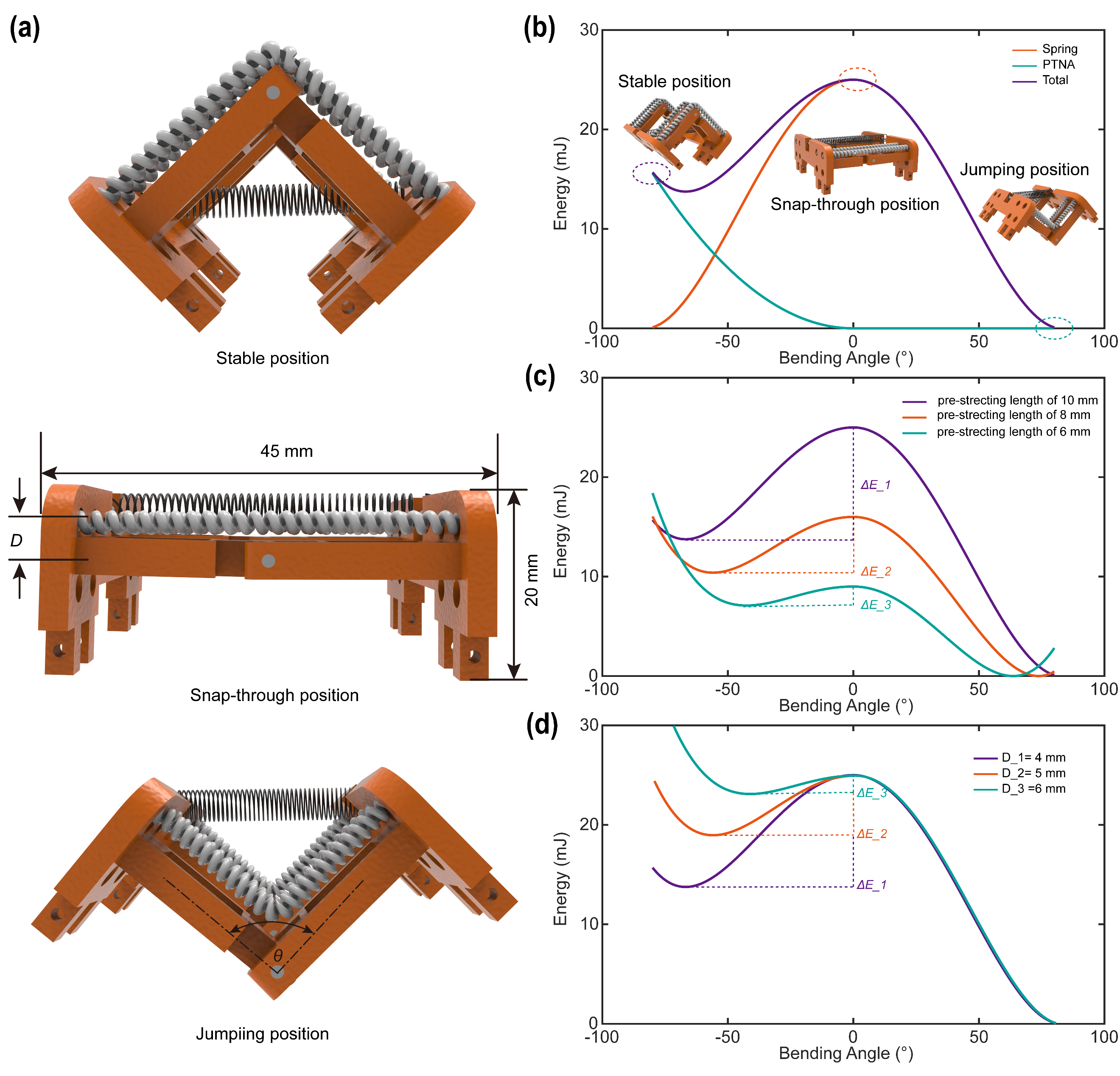


Fig. S10. Design of the small jumping robot. (A) Diagram of jumping. (B) Energy changes during the jumping motion. (C) Relationship between the energy change of the system and the pre-stretching length of spring. (D) Relationship between energy changes and distance between PTNA and robot center.


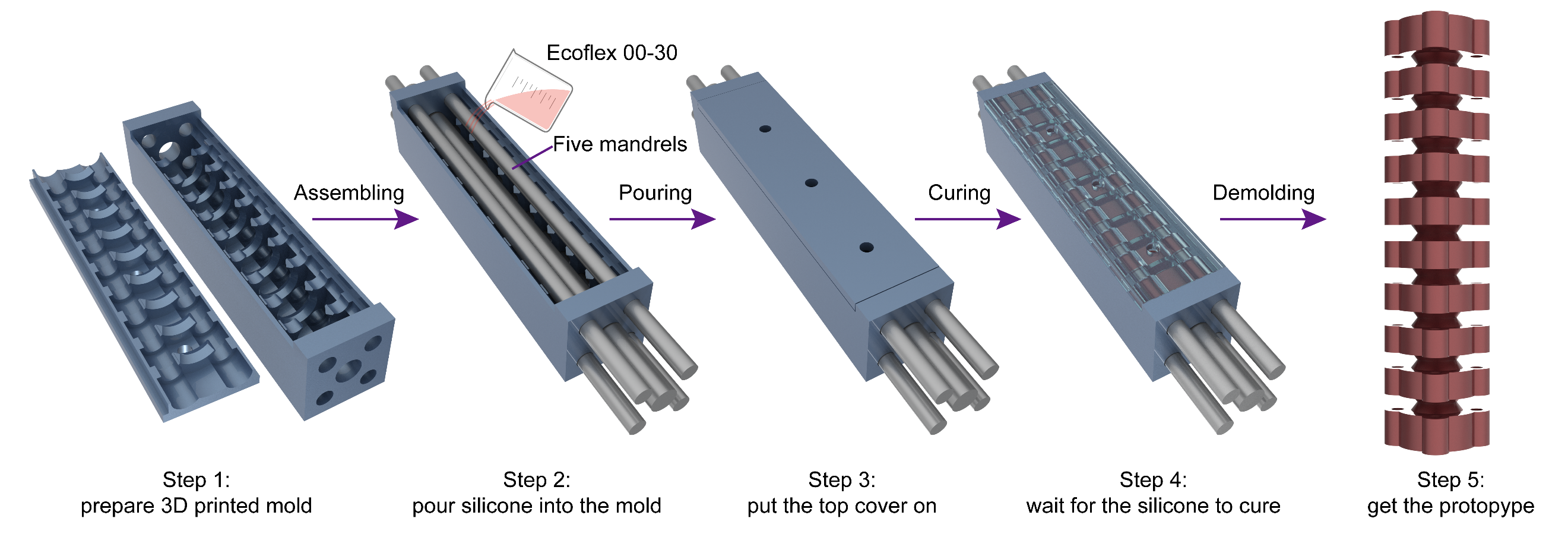


Fig. S11. Fabrication process of the 3-DOF soft finger.


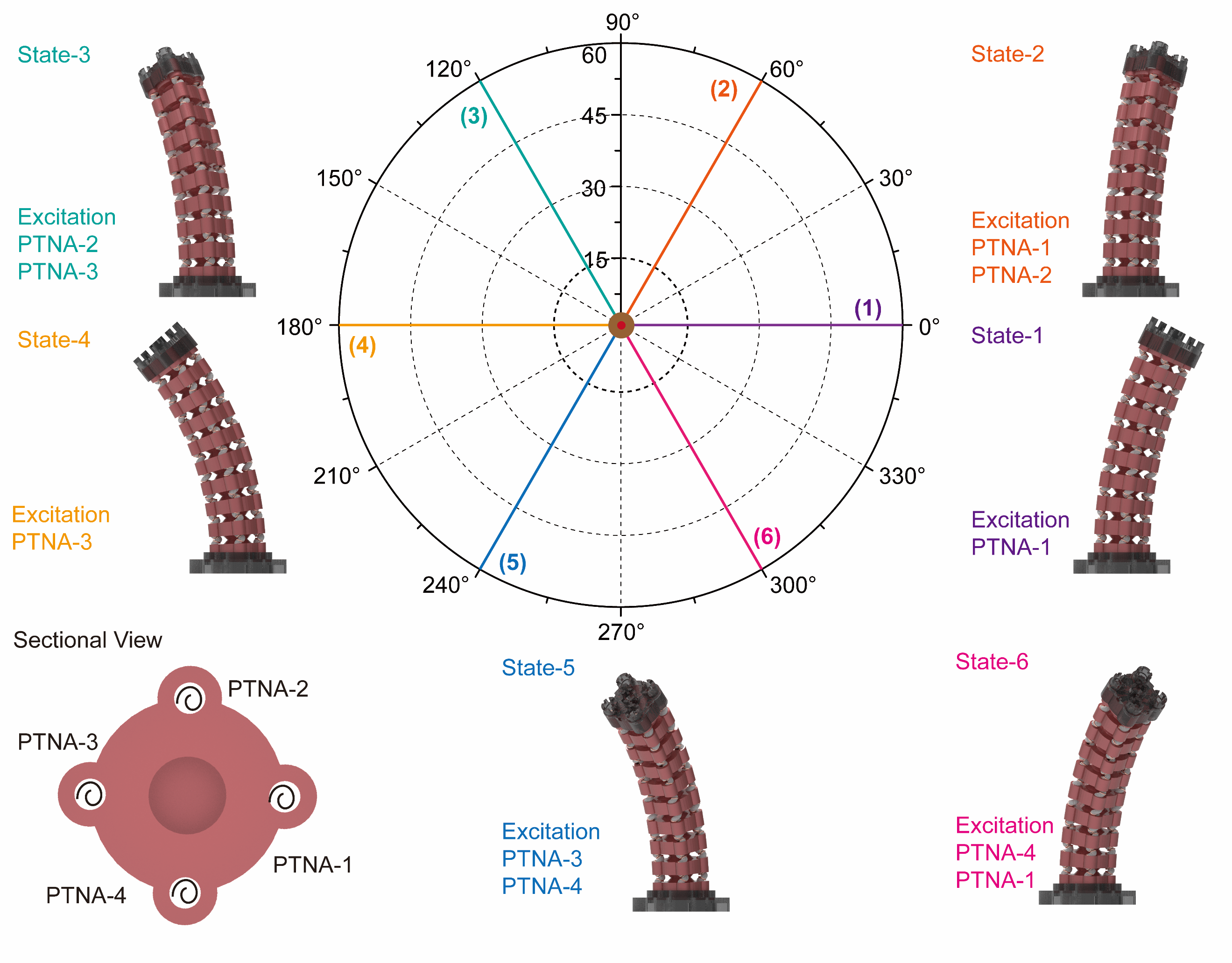


Fig. S12. Typical states for the bending motion of the soft finger.

**
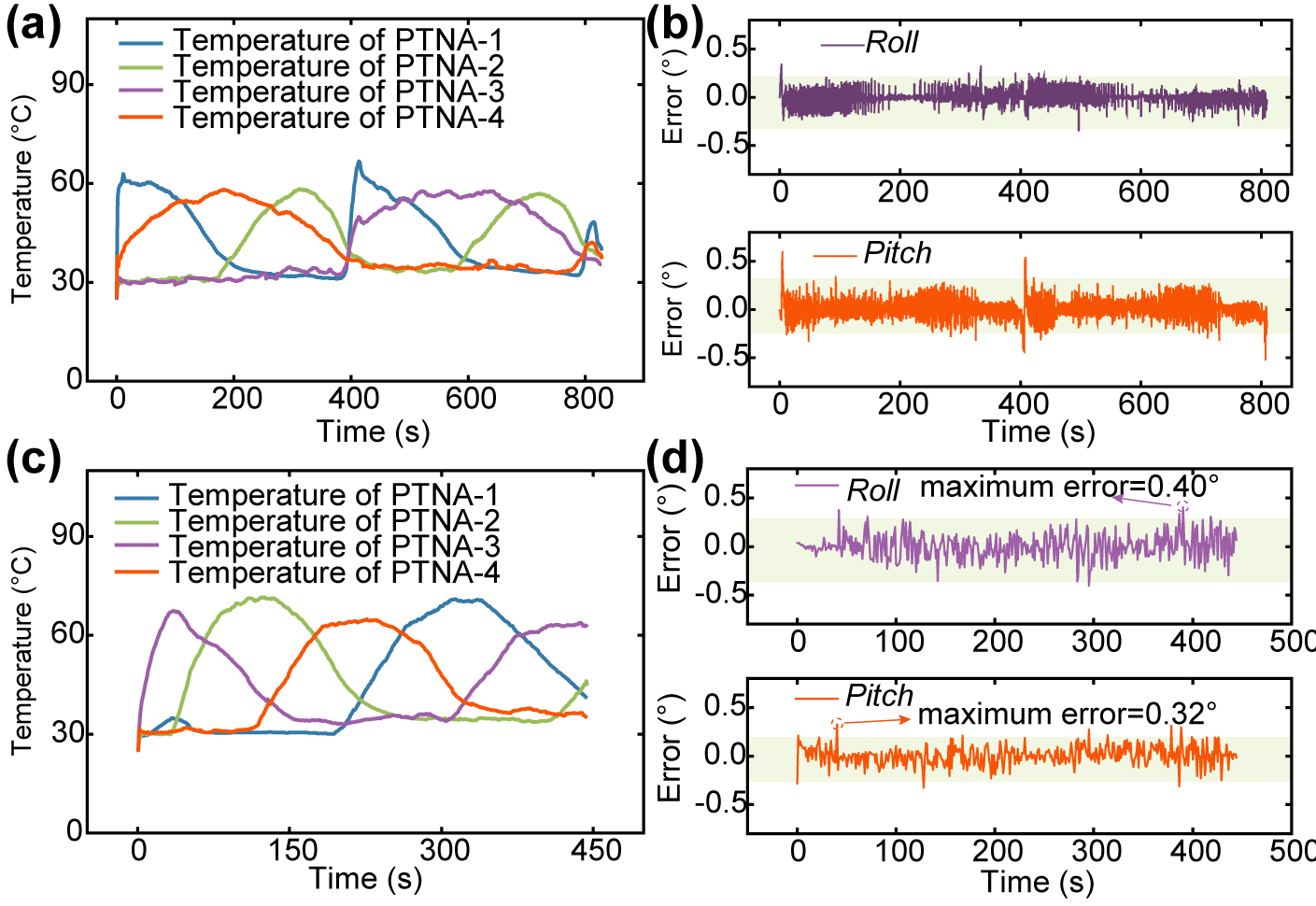
**

Fig. S13. Experimental results of temperature and errors for tracking different curves. (A-B) Temperature of PTNAs and tracking errors when tracking the ‘8’-like curve. (C-D) Temperature of PTNAs and tracking errors when tracking the ellipse curve.

Table S1. Main Material Parameters of the TNAs.

| Materials | Density (kg/m3) | Thermal conductivity (W/(m·°C)) | Convective heat  transfer coefficient  (W/(m2·°C)) |
| --- | --- | --- | --- |
| Nylon fiber | 1140 | 0.24 | 20 |
| Copper wire | 8933 | 80 | 10 |
