# Supplementary figures and images for: High-Performance Twisted Nylon Actuators for Soft Robots
Source: Research (Wash D C). 2023 Mar 17;8:0642. doi: 10.34133/research.0642 (PMC11912876; doi:10.34133/research.0642)

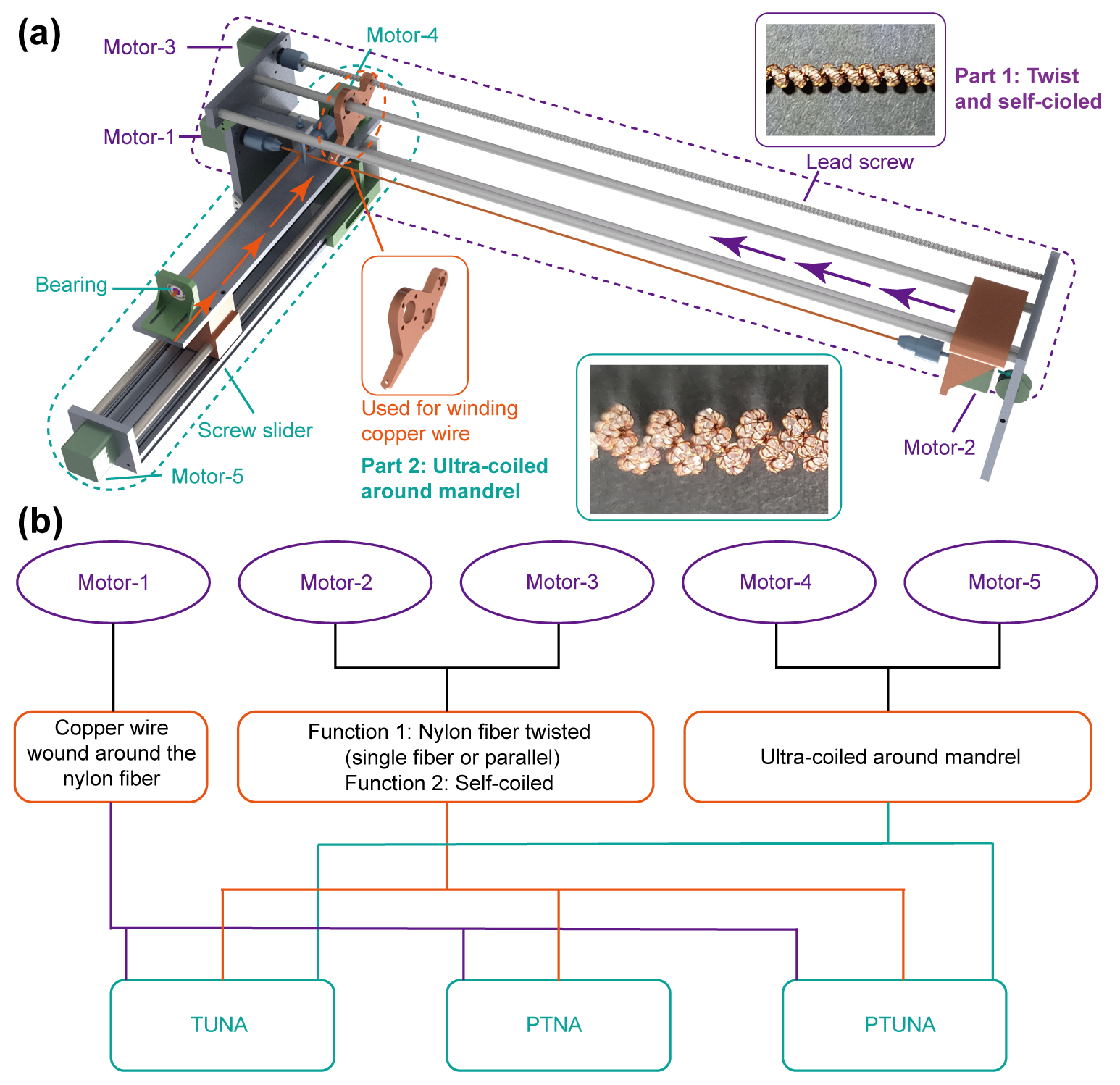

Supplement: Supplementary 1 — Notes S1 to S7 Figs. S1 to S13 Table S1 Movies S1 to S10 [file research.0642.f1.zip › Fig. S1.tif]

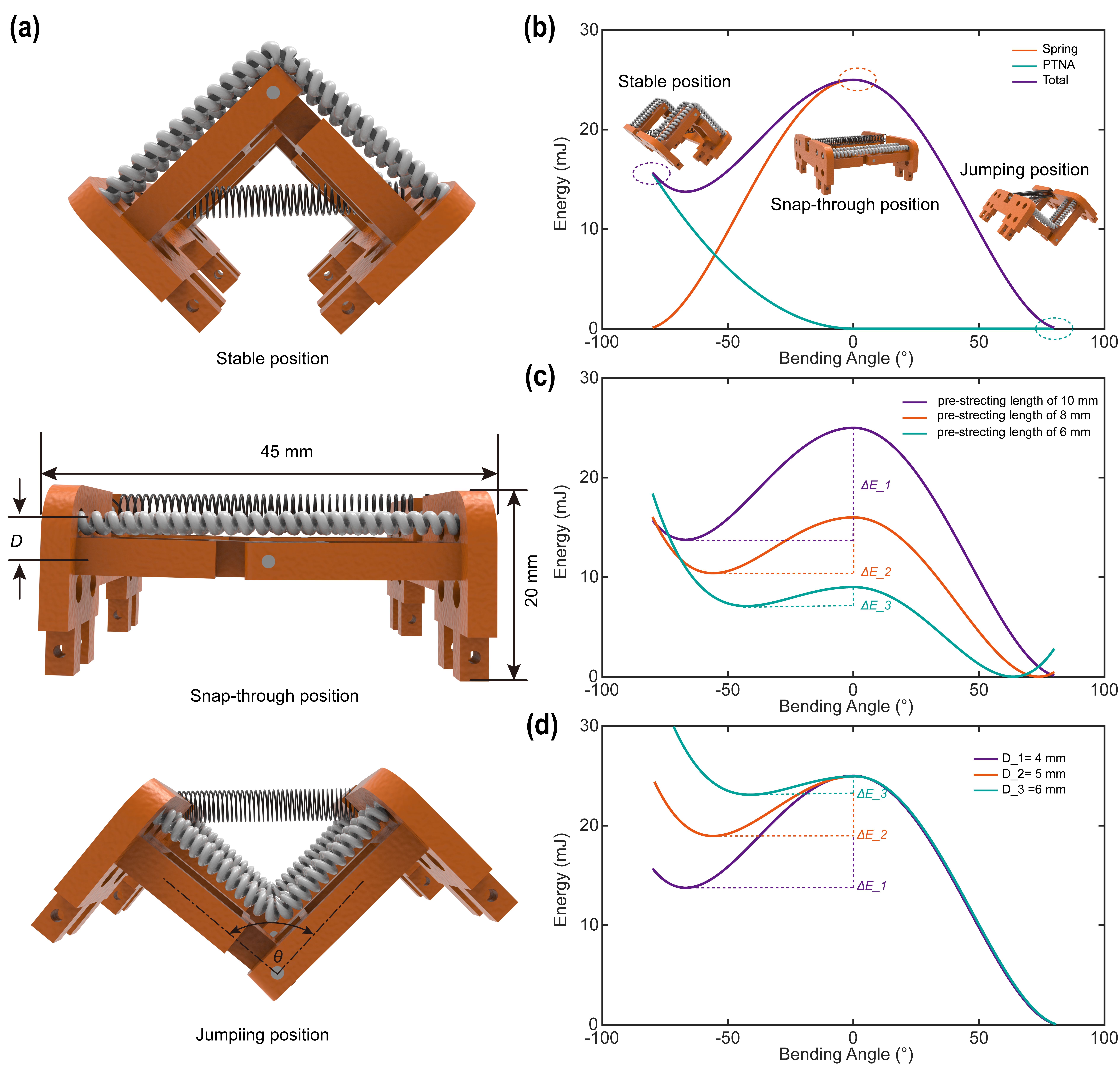

Supplement: Supplementary 1 — Notes S1 to S7 Figs. S1 to S13 Table S1 Movies S1 to S10 [file research.0642.f1.zip › Fig. S10.tif]

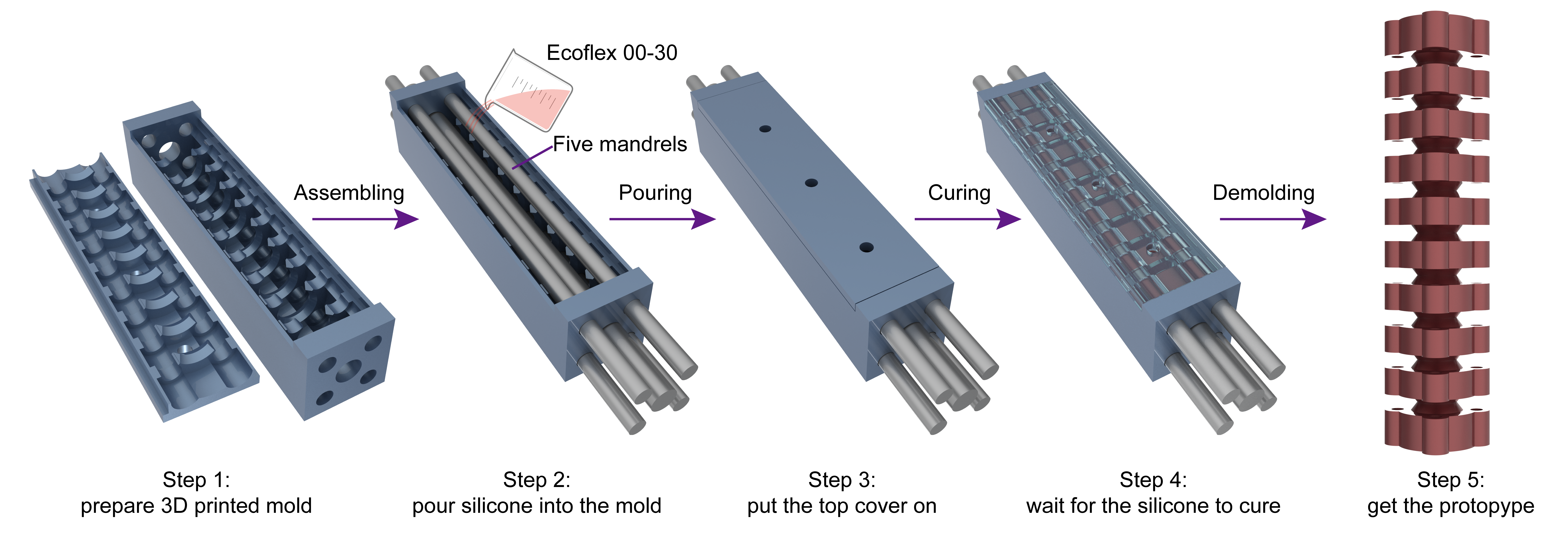

Supplement: Supplementary 1 — Notes S1 to S7 Figs. S1 to S13 Table S1 Movies S1 to S10 [file research.0642.f1.zip › Fig. S11.tif]

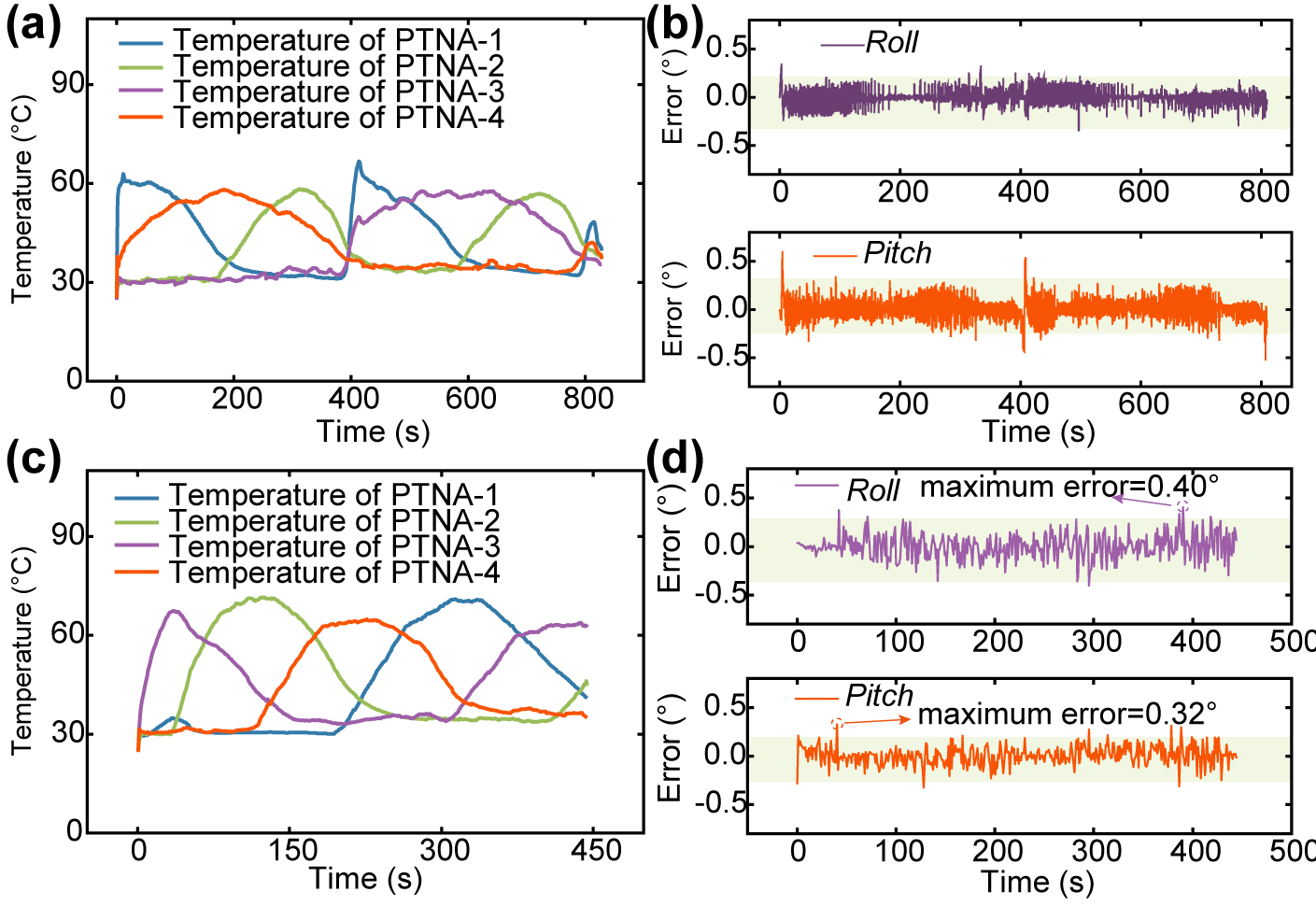

Supplement: Supplementary 1 — Notes S1 to S7 Figs. S1 to S13 Table S1 Movies S1 to S10 [file research.0642.f1.zip › Fig. S13.tif]

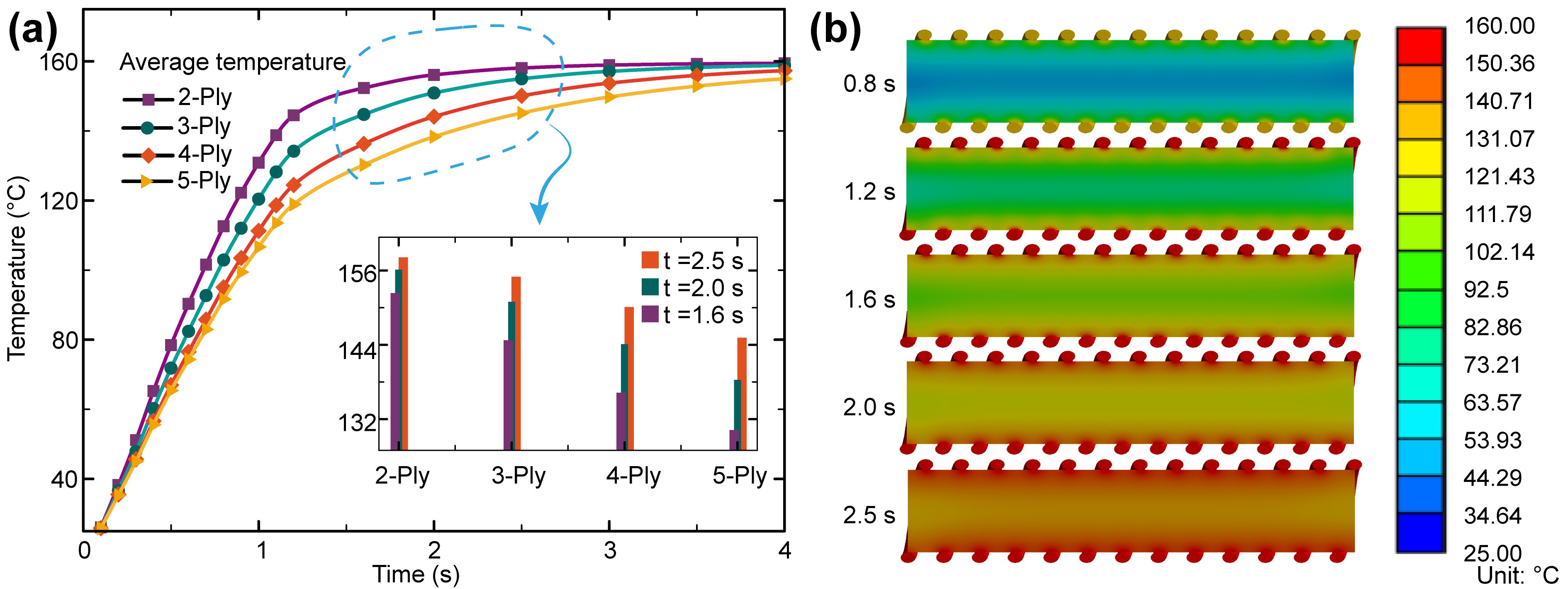

Supplement: Supplementary 1 — Notes S1 to S7 Figs. S1 to S13 Table S1 Movies S1 to S10 [file research.0642.f1.zip › Fig. S3.tif]

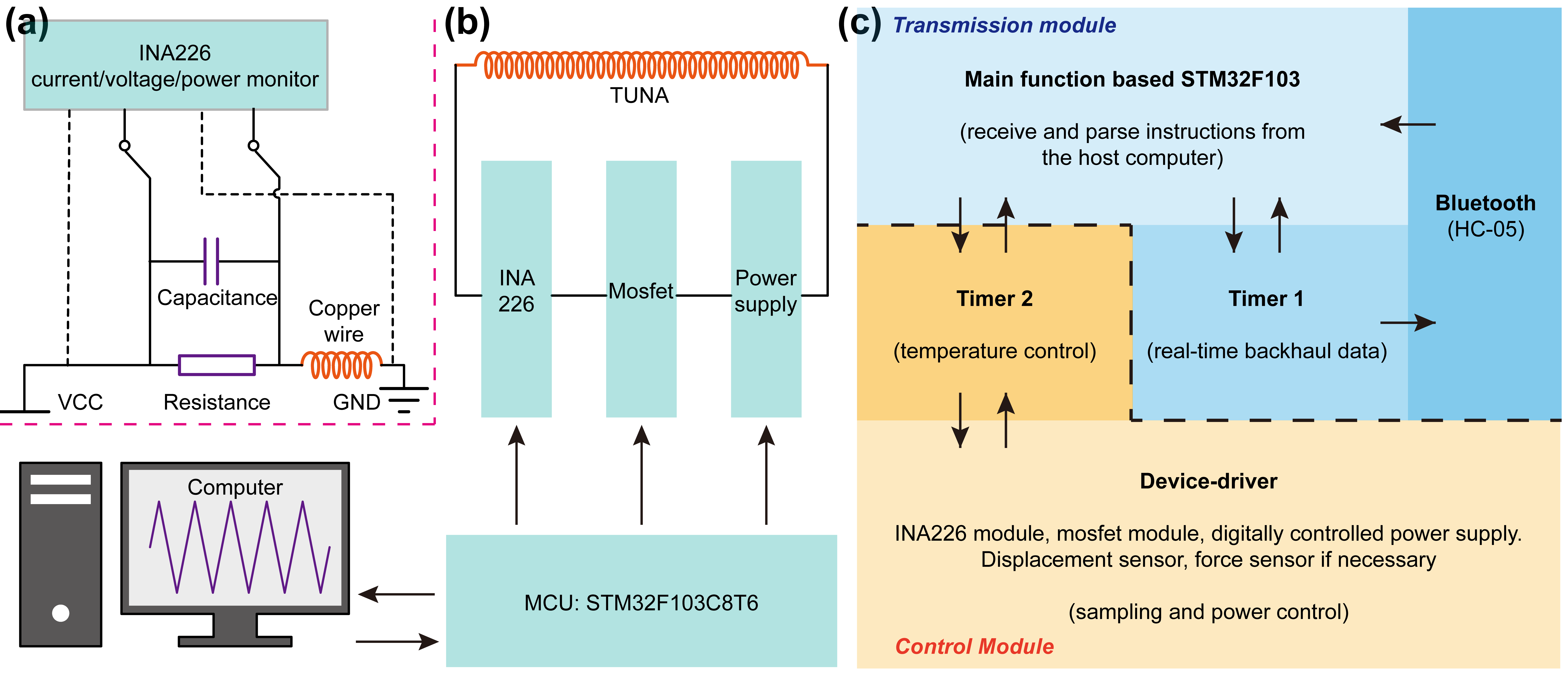

Supplement: Supplementary 1 — Notes S1 to S7 Figs. S1 to S13 Table S1 Movies S1 to S10 [file research.0642.f1.zip › Fig. S4.tif]

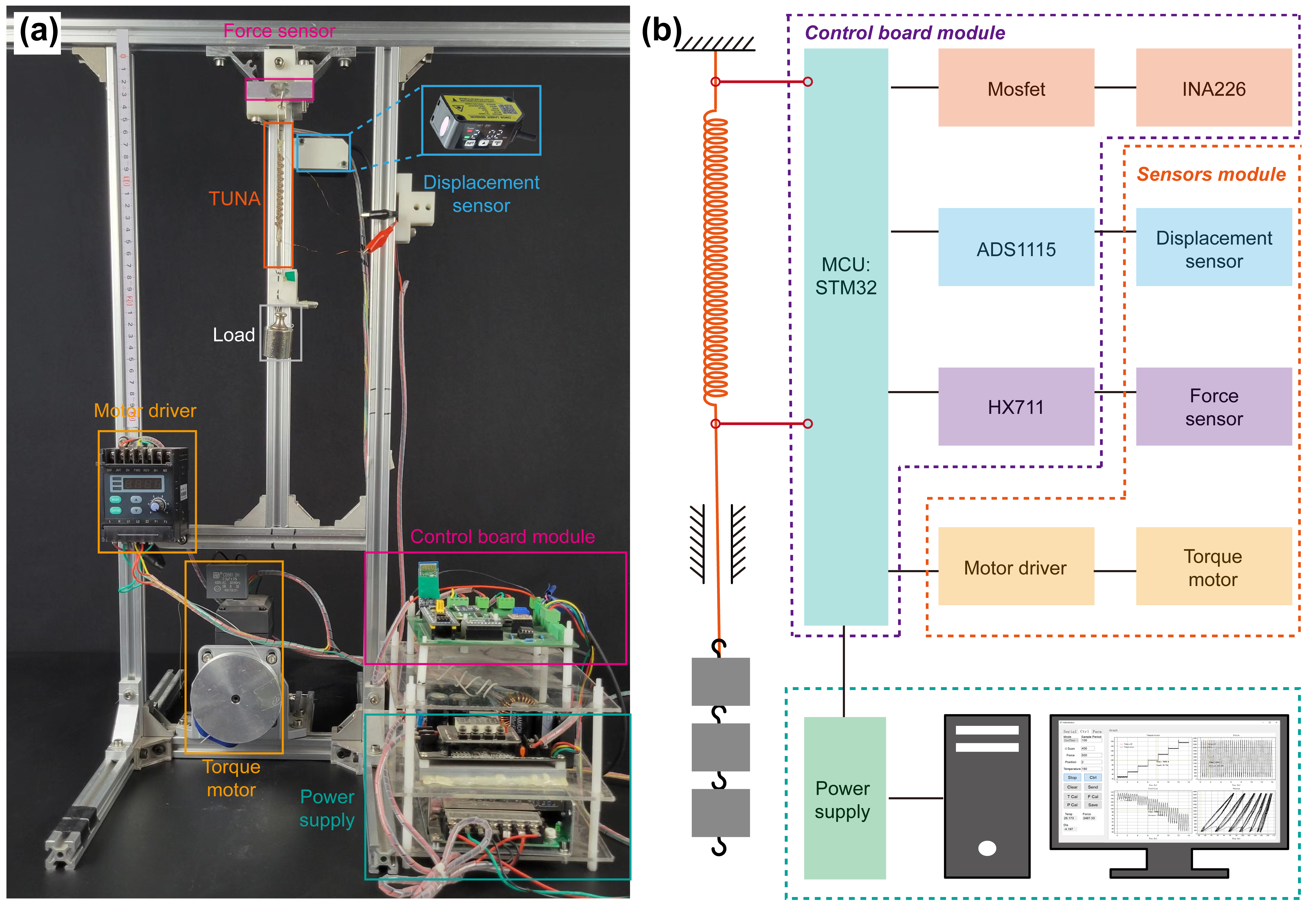

Supplement: Supplementary 1 — Notes S1 to S7 Figs. S1 to S13 Table S1 Movies S1 to S10 [file research.0642.f1.zip › Fig. S5.tif]

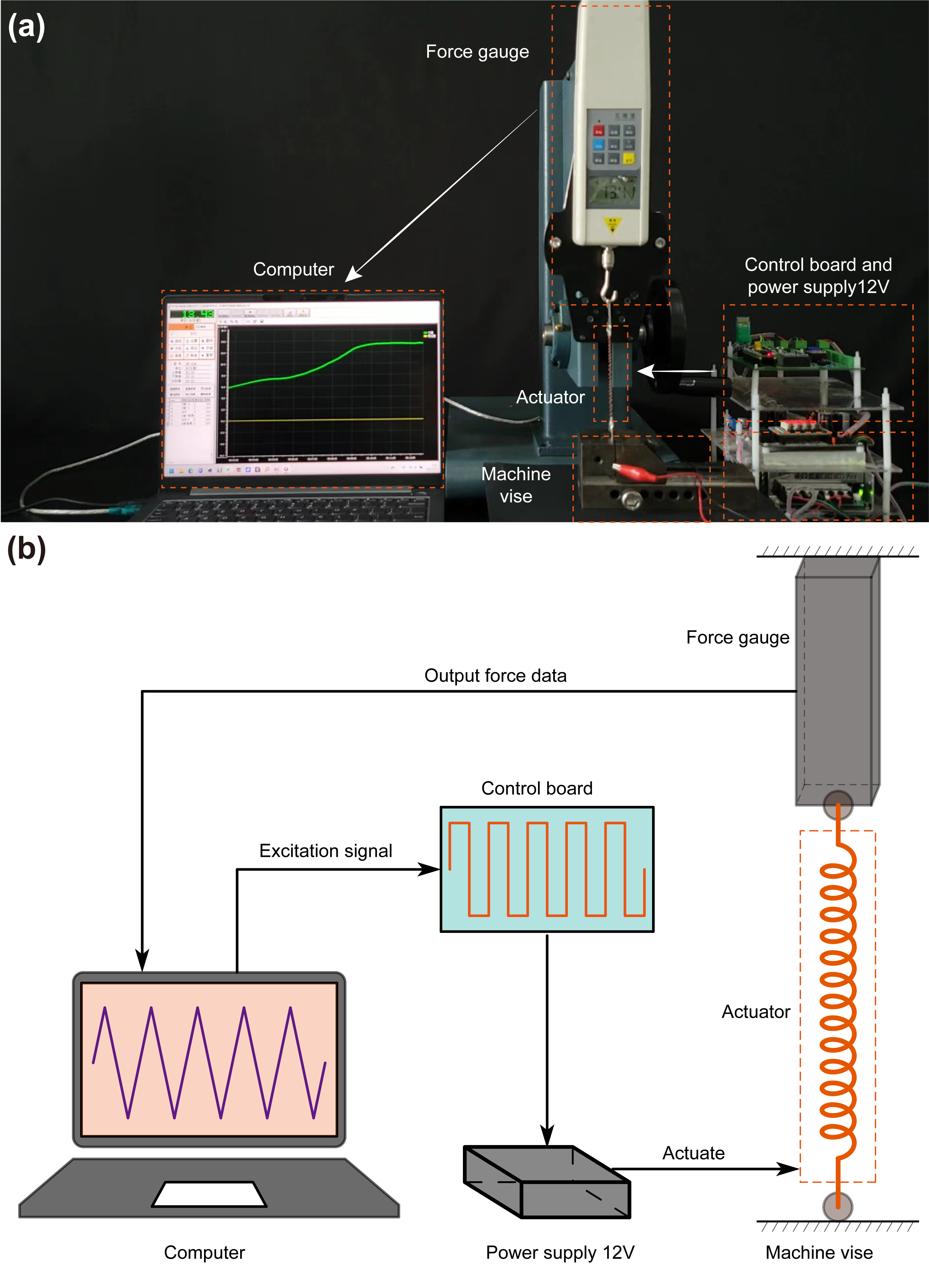

Supplement: Supplementary 1 — Notes S1 to S7 Figs. S1 to S13 Table S1 Movies S1 to S10 [file research.0642.f1.zip › Fig. S6.tif]

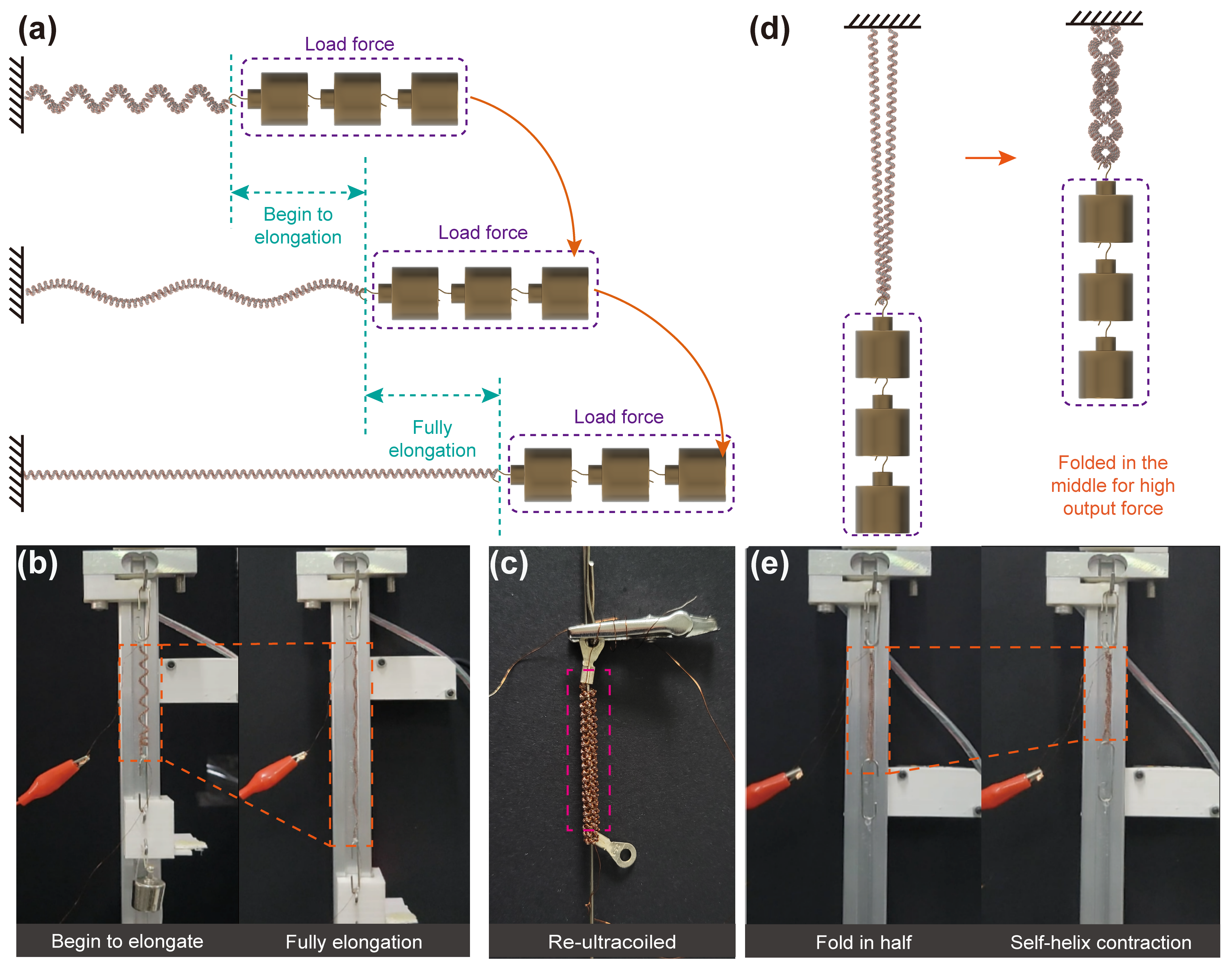

Supplement: Supplementary 1 — Notes S1 to S7 Figs. S1 to S13 Table S1 Movies S1 to S10 [file research.0642.f1.zip › Fig. S7.tif]

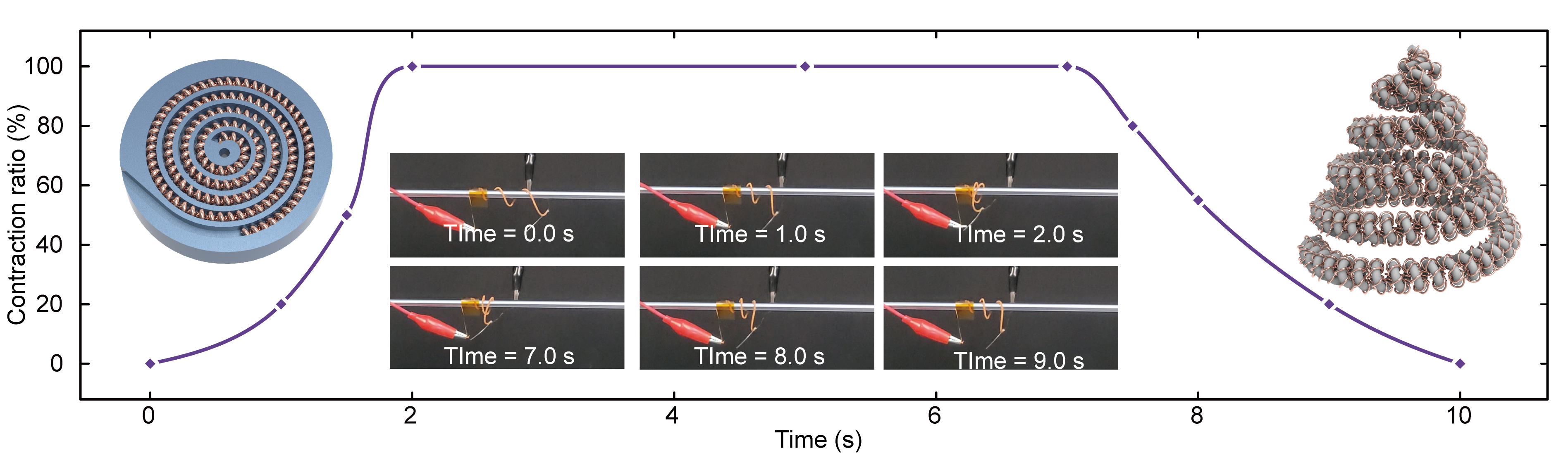

Supplement: Supplementary 1 — Notes S1 to S7 Figs. S1 to S13 Table S1 Movies S1 to S10 [file research.0642.f1.zip › Fig. S8.tif]

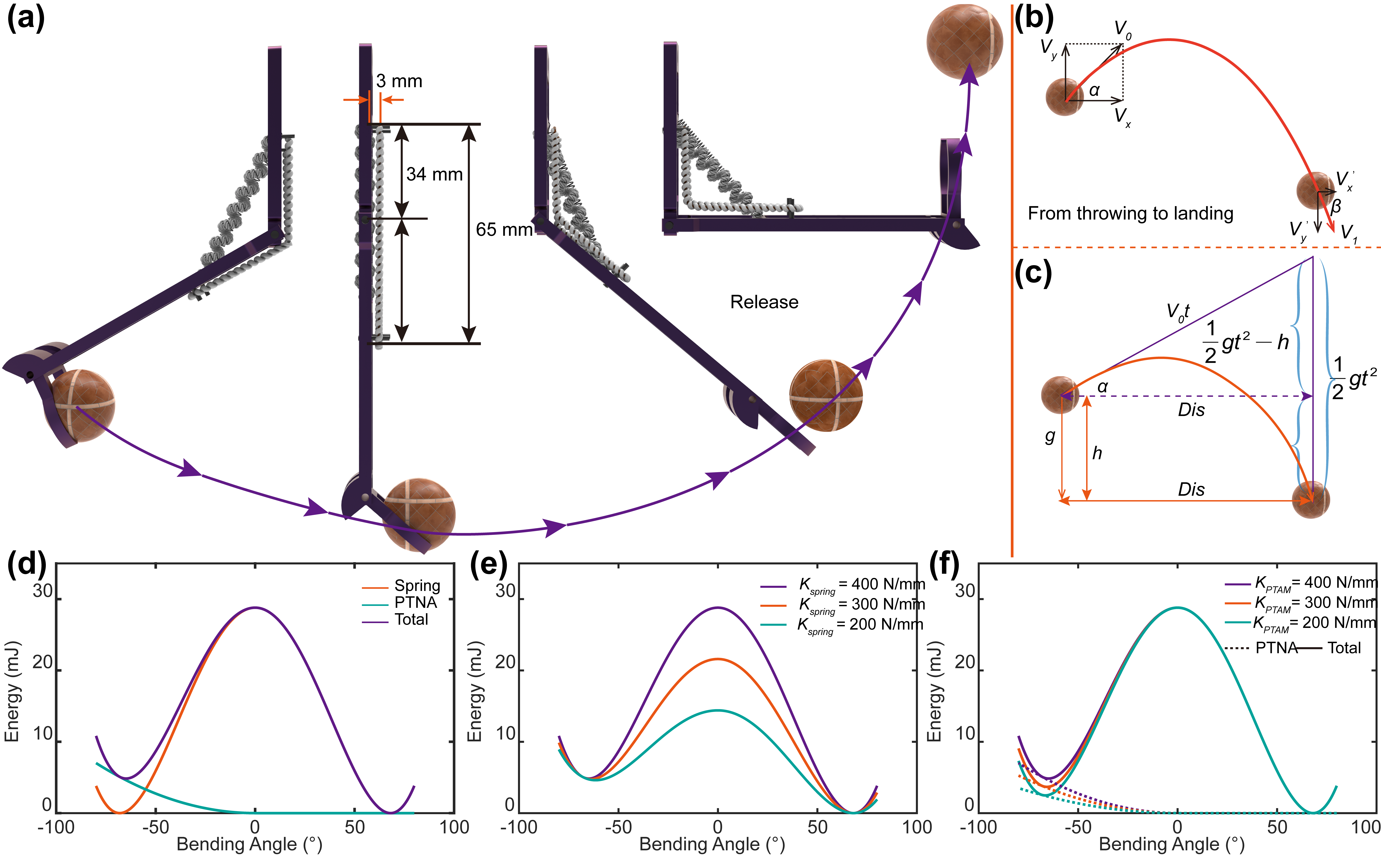

Supplement: Supplementary 1 — Notes S1 to S7 Figs. S1 to S13 Table S1 Movies S1 to S10 [file research.0642.f1.zip › Fig. S9.tif]
